# Prehospital predictors of clinical outcomes in out-of-hospital cardiac arrest with pulseless electrical activity: a JCS-ReSS report

**DOI:** 10.1016/j.resplu.2026.101508

**Published:** 2026-09-28

**Authors:** Shumpei Kosugi, Yasunori Ueda, Haruhiko Abe, Taro Takeuchi, Koichi Inoue, Yasushi Matsumura, Naohiro Yonemoto, Tetsuya Matoba, Yoshio Tahara

**Affiliations:** a Cardiovascular Division, National Hospital Organization Osaka National Hospital, 2-1-14 Hoenzaka, Chuo-ku, Osaka 540-0006, Japan; b Japanese Circulation Society Resuscitation Science Study (JCS-ReSS) Group, Japan

**Keywords:** Cardiopulmonary resuscitation, Electromechanical dissociation, Prognostication, Aetiology, Risk factors, Epidemiology

## Abstract

**Background:**

Out-of-hospital cardiac arrest (OHCA) with pulseless electrical activity (PEA) has a poor prognosis and is aetiologically heterogeneous. We aimed to identify prehospital predictors of a favourable outcome, overall and by presumed aetiology.

**Methods:**

This nationwide cohort study analysed 442,630 adults with OHCA and an initial rhythm of PEA from the All-Japan Utstein Registry between 2005 and 2024. The primary outcome was a favourable neurological outcome at 1 month. Two models were fitted, at the scene and at hospital arrival, each also within each aetiology. Epinephrine and advanced airway management (AAM) were assessed by time-dependent propensity score sequential matching.

**Results:**

At the scene, a witnessed arrest (risk ratio [RR], 1.65) predicted a favourable neurological outcome, and a longer response time predicted a worse one (RR, 0.66; 10 versus 5 min). The gradient with age was steeper in cardiac than non-cardiac PEA (RR, 0.38 versus 0.78). At hospital arrival, prehospital return of spontaneous circulation (RR, 23.31) was the strongest predictor, but conversion to a shockable rhythm predicted a favourable outcome only in cardiac PEA and a worse outcome in non-cardiac PEA (RR, 1.37 versus 0.73). Standardised to a common covariate distribution, 1-month survival was higher in non-cardiac PEA, whereas a favourable neurological outcome was more likely in cardiac PEA. After time-dependent adjustment, the apparent harm from epinephrine and AAM was attenuated.

**Conclusions:**

Prehospital information available before hospital arrival predicted a favourable neurological outcome, but its prognostic meaning depended on the presumed aetiology. These independent prehospital predictors could inform future risk stratification.

## Introduction

In out-of-hospital cardiac arrest (OHCA), the initial rhythm strongly predicts survival. Patients with shockable rhythms consistently demonstrate better outcomes than those with non-shockable rhythms, such as pulseless electrical activity (PEA) and asystole.^1^, ^2^, ^3^ The proportion of PEA among OHCA is rising,^4^, ^5^ so improving survival in PEA has become a primary objective of OHCA care.

Patients initially presenting with PEA consistently have higher survival rates than those with asystole, even though both are categorised as non-shockable rhythms.^6^, ^7^, ^8^ However, the PEA population is heterogeneous, including both candidates for aggressive interventions, such as mechanical circulatory support, and patients for whom such treatments would be inappropriate. Because decisions in the emergency setting rely on limited prehospital information, predictors of favourable outcomes using only these early data are needed, yet research on prehospital factors and prognosis in PEA remains scarce. Furthermore, much of this heterogeneity is aetiological. Arrests of presumed cardiac origin usually arise from a myocardial substrate and include potentially reversible causes such as acute coronary occlusion,^9^, ^10^ whereas arrests of non-cardiac origin are heterogeneous, ranging from acute external causes such as trauma, asphyxia and overdose to the terminal phase of an irreversible illness.^11^ Whether the prehospital factors that predict outcome differ between these two groups has not been established.

We therefore investigated the association between prehospital factors and outcomes in adults with OHCA initially presenting with PEA, in the cohort as a whole and separately according to the presumed aetiology of the arrest.

## Materials and methods

### Study design, setting, and population

This retrospective analysis was conducted using data from the All-Japan Utstein Registry, a prospectively maintained, population-based database managed by the Fire and Disaster Management Agency (FDMA) of Japan. We included all OHCA patients with PEA as the initial documented rhythm who were treated by emergency medical services (EMS) personnel and transported to a medical institution between January 1, 2005, and December 31, 2024. We excluded those with OHCA witnessed by EMS personnel or of unknown arrest status, and those younger than 18 years (Fig. S1). This study was approved by the Osaka National Hospital Institutional Review Board #2, and the requirement for individual informed consent was waived because the data were anonymised and registry-based.

In the Japanese EMS system, certified paramedics may perform advanced airway management (AAM) and administer epinephrine under online medical direction, and are generally not authorised to terminate resuscitation in the field. Resuscitation protocols followed the Japan Resuscitation Council guidelines of 2005, 2010, 2015, and 2020.^12^, ^13^, ^14^, ^15^

### Variables and outcomes

Data were extracted according to a standardised, Utstein-style template and comprised the variables listed in Table 1, together with the presumed aetiology of the arrest, recorded by the registry as cardiac or non-cardiac. Time-dependent variables included the response time (call to scene arrival), the post-arrival interval (scene arrival to hospital arrival), the interval from call to the first prehospital epinephrine administration and AAM, and the interval from call to prehospital return of spontaneous circulation (ROSC). The time of epinephrine administration has been recorded only since 2011, and the time of AAM only since 2021. The study period was categorised into four intervals (2005–2009, 2010–2014, 2015–2019, and 2020–2024) to align with major updates in resuscitation guidelines. Registry data were recorded on standardised forms by EMS personnel and cleansed for academic use by a subcommittee of the Japanese Circulation Society's resuscitation science study group (JCS-ReSS). The primary outcome was a favourable neurological outcome at 1 month, defined as a Cerebral Performance Category (CPC) of 1 or 2. The secondary outcome was survival at 1 month.

**Table 1:** Baseline characteristics of the study cohort, overall and by presumed aetiology.

		**Presumed aetiology**			
**Variable**	**Overall** **(*n* = 442,630)**	**Cardiac** **(*n* = 251,423)**	**Non-cardiac** **(*n* = 191,207)**	**Standardised difference**	**Missing** ***n* (%)**
**Patient characteristics**					
Age, years	80 [70,87]	81 [72,87]	78 [66,86]	0.29	0 (0.0)
Male	256,008 (57.8)	145,993 (58.1)	110,015 (57.5)	0.01	0 (0.0)
**Arrest circumstances**					
Witnessed by bystander	274,584 (62.0)	151,811 (60.4)	122,773 (64.2)	0.08	0 (0.0)
Bystander CPR	206,332 (46.6)	117,126 (46.6)	89,206 (46.7)	0.00	1 (0.0)
**Time intervals, min**					
Response time (call to scene arrival)	7 [6,9]	7 [6,9]	7 [5,9]	0.05	287 (0.1)
Post-arrival interval (scene arrival to hospital arrival)	24 [19,31]	24 [19,31]	24 [18,31]	0.02	565 (0.1)
No-flow time (collapse to first CPR)†	5 [1,11]	5 [1,11]	6 [1,11]	0.01	1110 (0.4)
**Resuscitation course**					
Conversion to shockable rhythm	28,065 (6.9)	20,520 (8.9)	7545 (4.3)	0.19	35,613 (8.0)
Physician-staffed ACLS	49,382 (11.2)	26,216 (10.4)	23,166 (12.1)	0.05	55 (0.0)
Prehospital epinephrine	122,638 (29.3)	74,731 (31.6)	47,907 (26.4)	0.11	24,647 (5.6)
Advanced airway management: none	203,193 (49.1)	110,108 (46.8)	93,085 (52.1)	0.11	28,566 (6.5)
Endotracheal intubation	39,242 (9.5)	19,217 (8.2)	20,025 (11.2)	0.10	28,566 (6.5)
Extraglottic airway	152,598 (36.9)	94,064 (40.0)	58,534 (32.7)	0.15	28,566 (6.5)
Laryngeal mask airway	19,031 (4.6)	11,853 (5.0)	7178 (4.0)	0.05	28,566 (6.5)
Prehospital ROSC	64,216 (14.5)	29,841 (11.9)	34,375 (18.0)	0.17	0 (0.0)
**Outcomes**					
Favourable neurological outcome at 1 month	9348 (2.1)	5513 (2.2)	3835 (2.0)	0.01	0 (0.0)
Survival at 1 month	28,063 (6.3)	13,278 (5.3)	14,785 (7.7)	0.10	0 (0.0)

Data are median [interquartile range] or *n* (%); percentages are calculated among cases with non-missing data, and the Missing column refers to the whole cohort.

ACLS, advanced cardiovascular life support; CPR, cardiopulmonary resuscitation; ROSC, return of spontaneous circulation.

† Bystander-witnessed arrests only (*n* = 274,584).

### Statistical analysis

Two multivariable logistic regression models were prespecified. Model A included only what is known to the EMS at the scene: age, sex, bystander witness, bystander cardiopulmonary resuscitation (CPR), the response time, and the study period. Model B added the course of the resuscitation up to hospital arrival: conversion to a shockable rhythm, prehospital ROSC, the post-arrival interval, and physician-staffed ACLS. The two models were reported side by side rather than combined, because the variables added in Model B lie downstream of those in Model A. Prehospital epinephrine and AAM were not covariates in either model but were examined separately below. Age, the response time, and the post-arrival interval were modelled with restricted cubic splines. Both models were also fitted separately within each aetiology, with the spline knots fixed at the whole-cohort quartiles so that the curves remain comparable; modification is reported on both the ratio and the difference scale (Tables S1 and S2). To compare the aetiologies themselves, each stratum-specific model was applied to the covariate distribution of the whole cohort and the standardised risks contrasted (Table S3). The primary effect measures were the marginal risk ratio (RR) and risk difference (RD), obtained by model-based marginal standardisation from the fitted models. Missing prehospital covariates were assumed to be missing at random and handled using multiple imputation by chained equations (30 datasets, 10 iterations; logistic regression for binary and predictive mean matching for continuous variables), and estimates were combined using Rubin’s rules. Outcomes and treatment indicators were not imputed, but outcomes were allowed as predictors in the imputation models. For epinephrine, we applied time-dependent propensity score sequential matching within 2011–2024, from EMS scene arrival to 59 min. At each minute, patients receiving their first dose were matched 1:1 with replacement to concurrent untreated patients still at risk, based on a propensity score estimated from a pooled logistic model with time-fixed baseline covariates. Confidence intervals (CIs) used cluster-robust variances at the patient level, and person-minutes after the end of prehospital care or after prehospital ROSC were excluded (Table S4). Because AAM timing has been recorded only since 2021, the matching could be applied to AAM only within 2021–2024; the landmark analysis at 10 min and the stabilised inverse probability of treatment weighting (IPTW) analysis were used as supporting analyses covering the whole study period. All intervention analyses, including the conventional multivariable estimate shown for reference, adjusted for the Model B covariates other than prehospital ROSC and the post-arrival interval, which lie downstream of treatment, with the presumed aetiology added. Covariate balance was assessed using standardised mean differences (Tables S5–S7), and all intervention analyses were repeated within each aetiology. Sensitivity analyses were performed by repeating all analyses using only complete cases, and by repeating them within each study period. Given the size of the cohort, associations are described by the magnitude of the estimate and the width of its CI rather than by statistical significance. All analyses were two-sided with 95% CIs and were conducted in R version 4.6.1 (The R Foundation for Statistical Computing, Vienna, Austria).

## Results

### Patient selection and characteristics

A total of 2,482,808 patients with OHCA were documented. After exclusions, 442,630 patients with an initial rhythm of PEA were analysed (Fig. S1). The baseline characteristics of the study cohort are presented in Table 1. The presumed aetiology was cardiac in 251,423 patients (56.8%) and non-cardiac in 191,207 (43.2%); those with a cardiac aetiology were older (median 81 versus 78 years) and more often converted to a shockable rhythm (8.9% versus 4.3%), whereas prehospital ROSC was more frequent with a non-cardiac aetiology (18.0% versus 11.9%). Overall, 9348 patients (2.1%) achieved a favourable neurological outcome, and 28,063 (6.3%) survived at 1 month; both proportions rose until 2015–2019 and fell in 2020–2024 (Table S8).

### Prehospital predictors of clinical outcomes

Fig. 1 shows the estimates for all covariates in both models. In Model A, a witnessed arrest was most strongly associated with a favourable neurological outcome (RR, 1.65) and increasing age with a lower probability of one (RR, 0.50 for 80 versus 60 years), as illustrated by the spline curve (Fig. 2A). Bystander CPR showed little association with the outcome (RR, 0.98; 95% CI, 0.94–1.02). In Model B, prehospital ROSC was by far the strongest predictor (RR, 23.31), and conversion to a shockable rhythm also predicted a favourable outcome (RR, 1.51). Longer response and post-arrival intervals were associated with worse outcomes in both models (Fig. 2B and C). Results for 1-month survival were consistent in direction but generally closer to the null (Figs. S2 and S3; Tables S9 and S10).

**Fig. 1:**
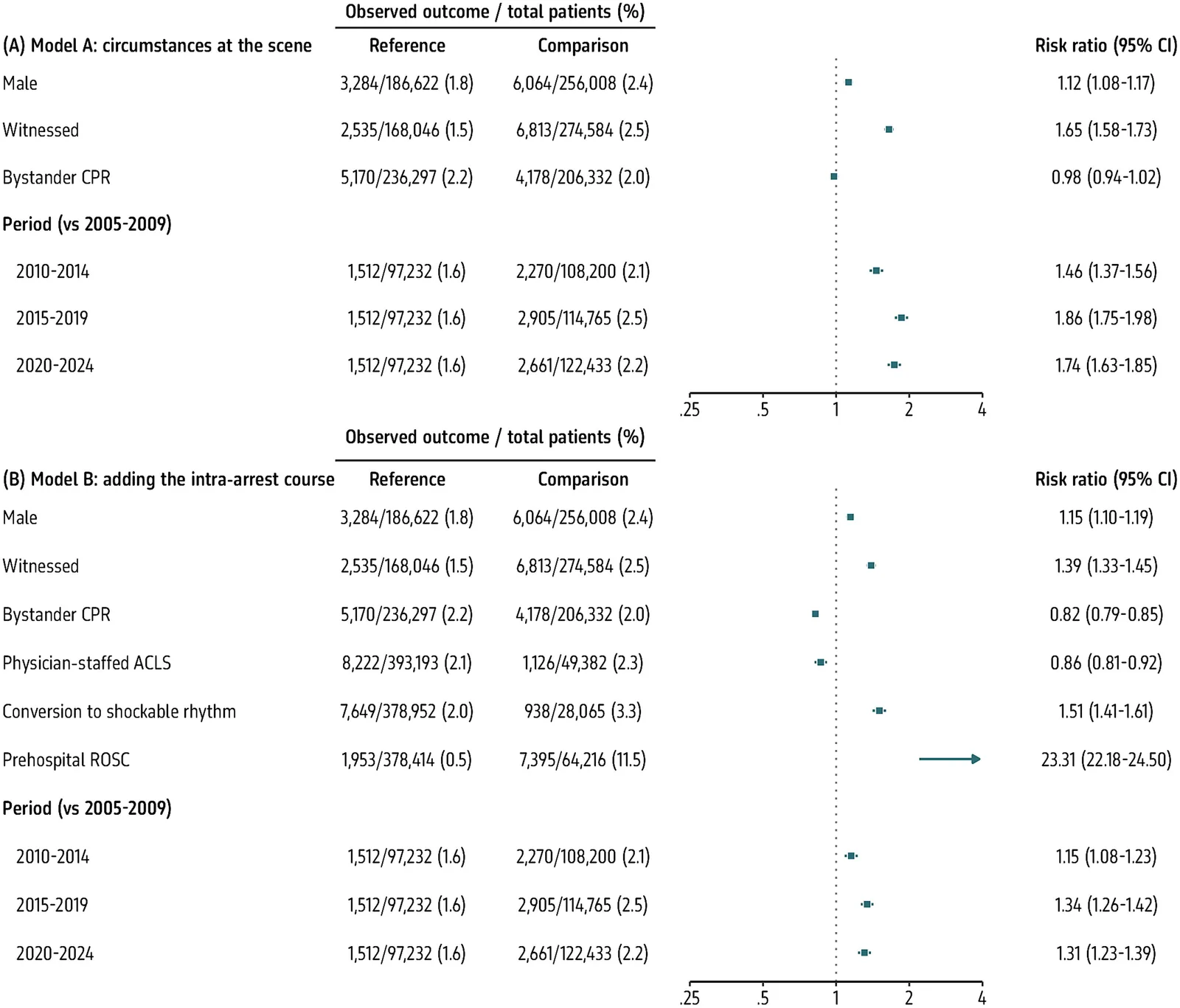
**Observed favourable neurological outcome rates and adjusted risk ratios associated with prehospital factors**. Observed rates of 1-month favourable neurological outcome (unadjusted counts/percentages) and adjusted risk ratios (RRs) with 95% confidence intervals for each prehospital factor, from (A) Model A and (B) Model B. The RRs were derived from a multivariable logistic regression model. Estimates outside the plotted range are shown as arrows. *ACLS, advanced cardiovascular life support; CPR, cardiopulmonary resuscitation; ROSC, return of spontaneous circulation.*

**Fig. 2:**
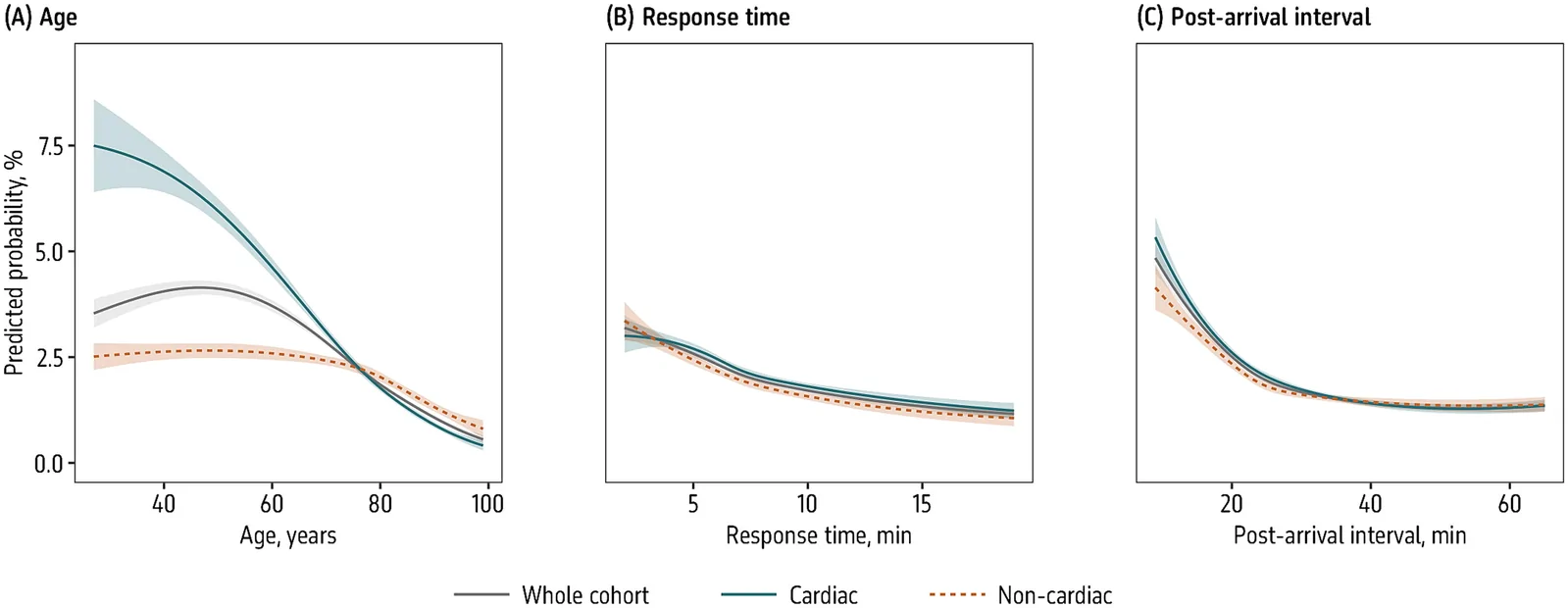
**Spline-based adjusted probabilities of favourable neurological outcome by age and prehospital time**. Predicted probabilities of a favourable neurological outcome at 1 month according to (A) age, (B) response time, and (C) the post-arrival interval, modelled using restricted cubic splines. The solid lines represent the adjusted probabilities, and the shaded areas represent the 95% confidence intervals. Each panel shows the whole cohort and the same variable estimated separately among patients with a cardiac and a non-cardiac aetiology.

### Predictors and outcomes by presumed aetiology

Fitting the two models separately within each aetiology changed the direction of several associations (Fig. 3). Conversion to a shockable rhythm predicted a favourable neurological outcome in cardiac PEA (RR, 1.37) but a worse outcome in non-cardiac PEA (RR, 0.73), and the same reversal was present for 1-month survival (RR, 1.33 versus 0.74). The male advantage was likewise confined to cardiac PEA (RR, 1.20 versus 0.97 for a favourable neurological outcome, and 1.11 versus 0.91 for survival). The gradient with age was steeper in cardiac PEA (RR, 0.38 versus 0.78), and for 1-month survival there was little relationship with age in non-cardiac PEA (RR, 1.00 versus 0.55). Prehospital ROSC remained the strongest predictor in both groups (RR, 26.54 and 20.39; Table S1), but the predicted probability of a favourable neurological outcome among those achieving it was 14.3% with a cardiac and 9.7% with a non-cardiac aetiology. The corresponding results for 1-month survival are shown in Fig. S4 and Table S2. Under a common covariate distribution, the standardised probability of a favourable neurological outcome was higher for cardiac PEA (2.38% versus 1.94%), whereas standardised 1-month survival was higher for non-cardiac PEA (7.55% versus 5.61%) (Table S3). Among 1-month survivors, 41.5% with a cardiac aetiology and 25.9% with a non-cardiac aetiology had a favourable neurological outcome (Table 1).

**Fig. 3:**
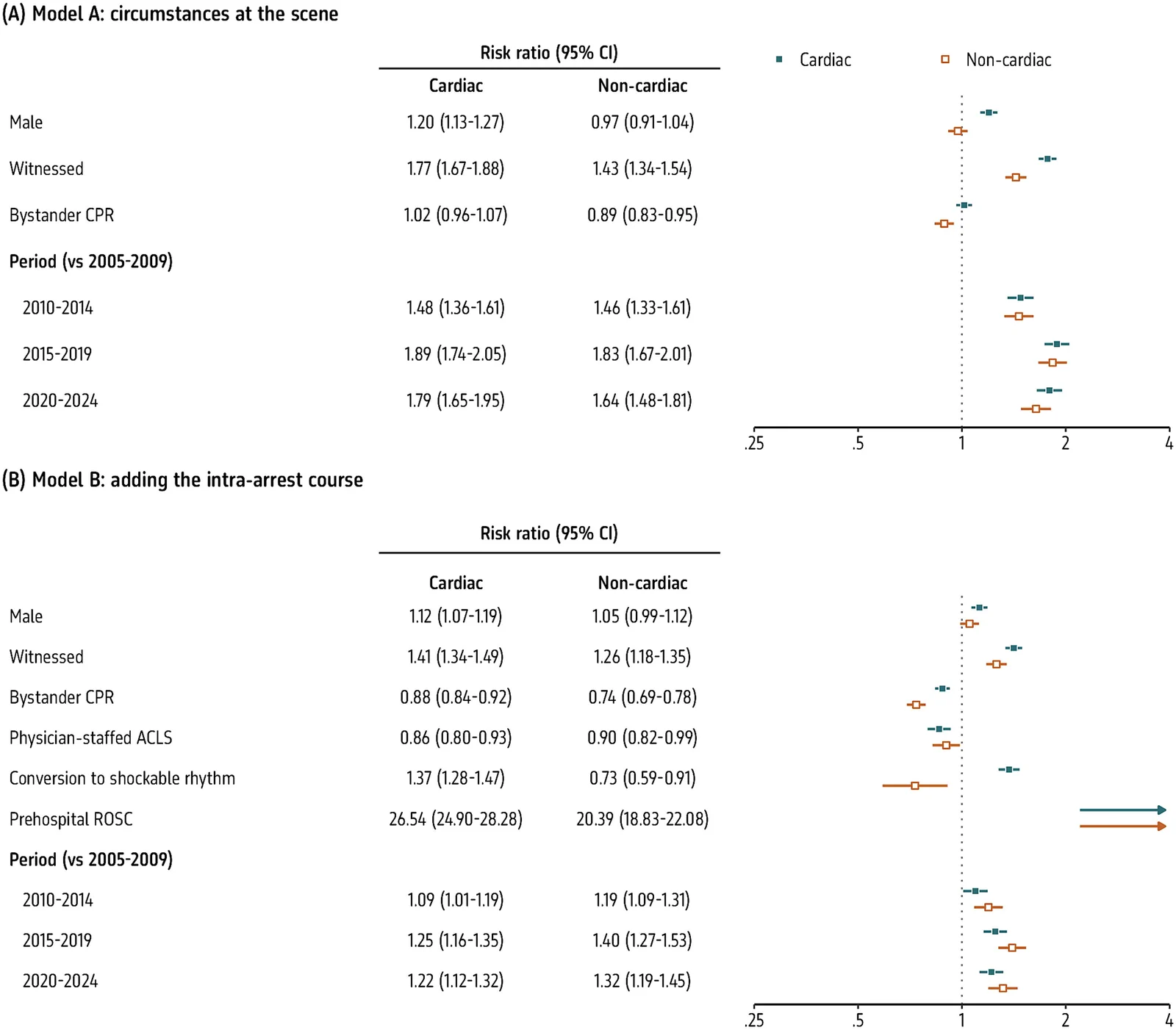
**Adjusted risk ratios for favourable neurological outcome, by presumed aetiology**. Risk ratios (RRs) with 95% confidence intervals for each prehospital factor, estimated separately among patients with a cardiac and a non-cardiac aetiology, from (A) Model A and (B) Model B. Filled squares are cardiac and open squares non-cardiac arrests; the two markers on each line are offset vertically. Estimates outside the plotted range are shown as arrows. *ACLS, advanced cardiovascular life support; CPR, cardiopulmonary resuscitation; ROSC, return of spontaneous circulation.*

### Effect of prehospital epinephrine administration

Propensity score sequential matching to adjust for time-dependent confounding created 109,172 matched pairs with excellent covariate balance (Table S5). The adjusted RR for a favourable neurological outcome was 1.00 (95% CI, 0.88–1.14), and for 1-month survival was 1.46 (95% CI, 1.37–1.56) (Fig. 4A). Most matched administrations (78.1%) occurred within 6–20 min of EMS arrival (Fig. 4B). The association with 1-month survival was present in both aetiologies, with a larger absolute benefit in non-cardiac PEA (Table S11).

**Fig. 4:**
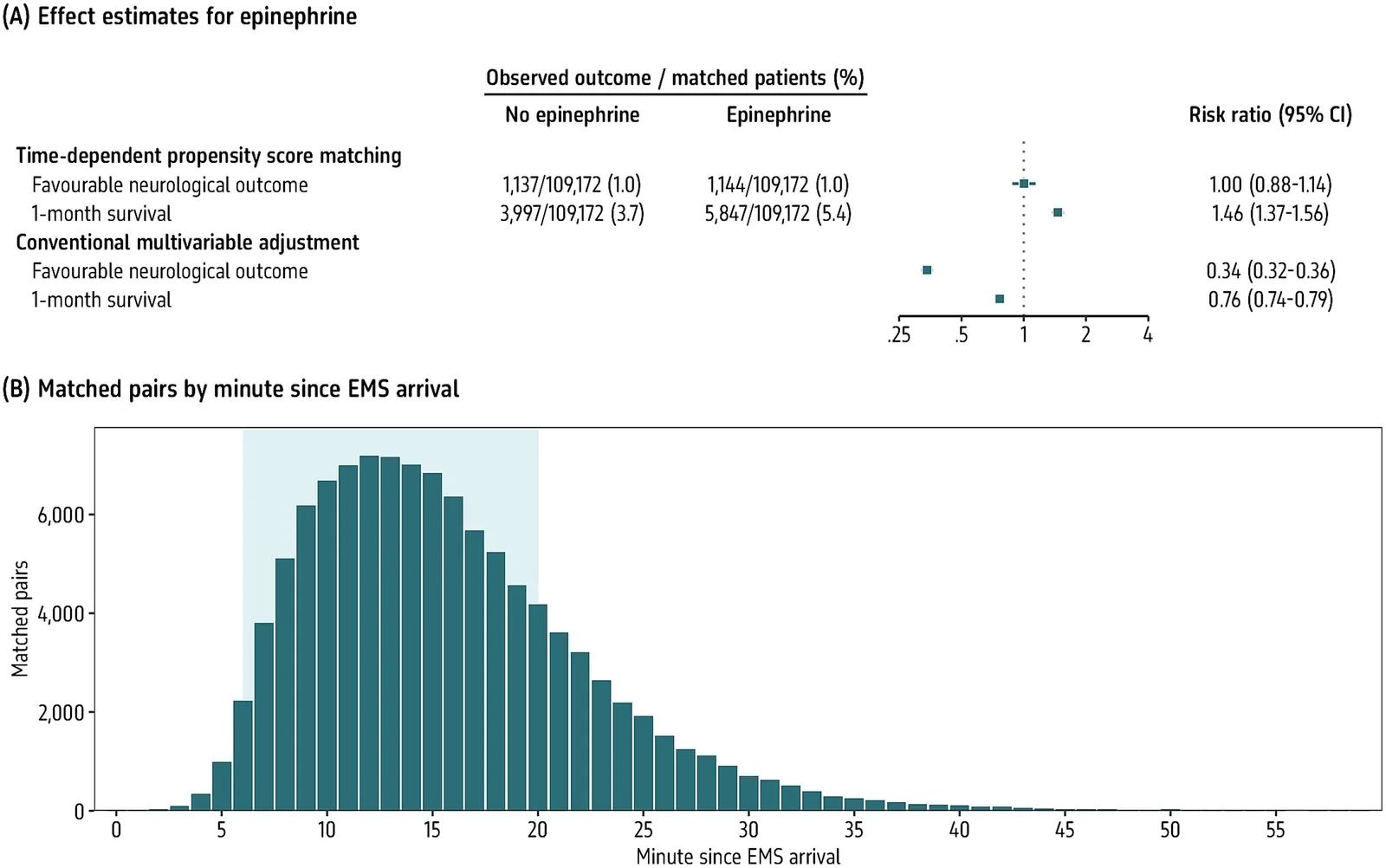
**Association of prehospital epinephrine with clinical outcomes using time-dependent propensity score matching**. (A) Forest plot showing the adjusted risk ratios (RRs) with 95% confidence intervals for the association between prehospital epinephrine administration and 1-month outcomes. Observed outcome rates (unadjusted counts/percentages) are shown for the matched epinephrine and no-epinephrine groups; the conventional multivariable estimate is not matched and therefore has no counts. (B) Histogram showing the distribution of matched pairs by the minute since emergency medical services arrival at which the first dose of epinephrine was administered. The shaded area highlights the 6-to-20-min interval, where 78.1% of matches occurred.

### Effect of prehospital AAM

The same matching approach applied to AAM within 2021–2024 (50,753 matched pairs; Table S6) showed a higher probability of 1-month survival (RR, 1.14) and a lower probability of a favourable neurological outcome (RR, 0.72) (Fig. 5A); 93.3% of matched placements occurred within 20 min of EMS arrival (Fig. 5C). Both estimates rose as the design better respected the timing of treatment, from conventional adjustment and weighting through the landmark analysis to time-dependent matching (Fig. 5A). Estimates by airway device are shown in Fig. 5B.

**Fig. 5:**
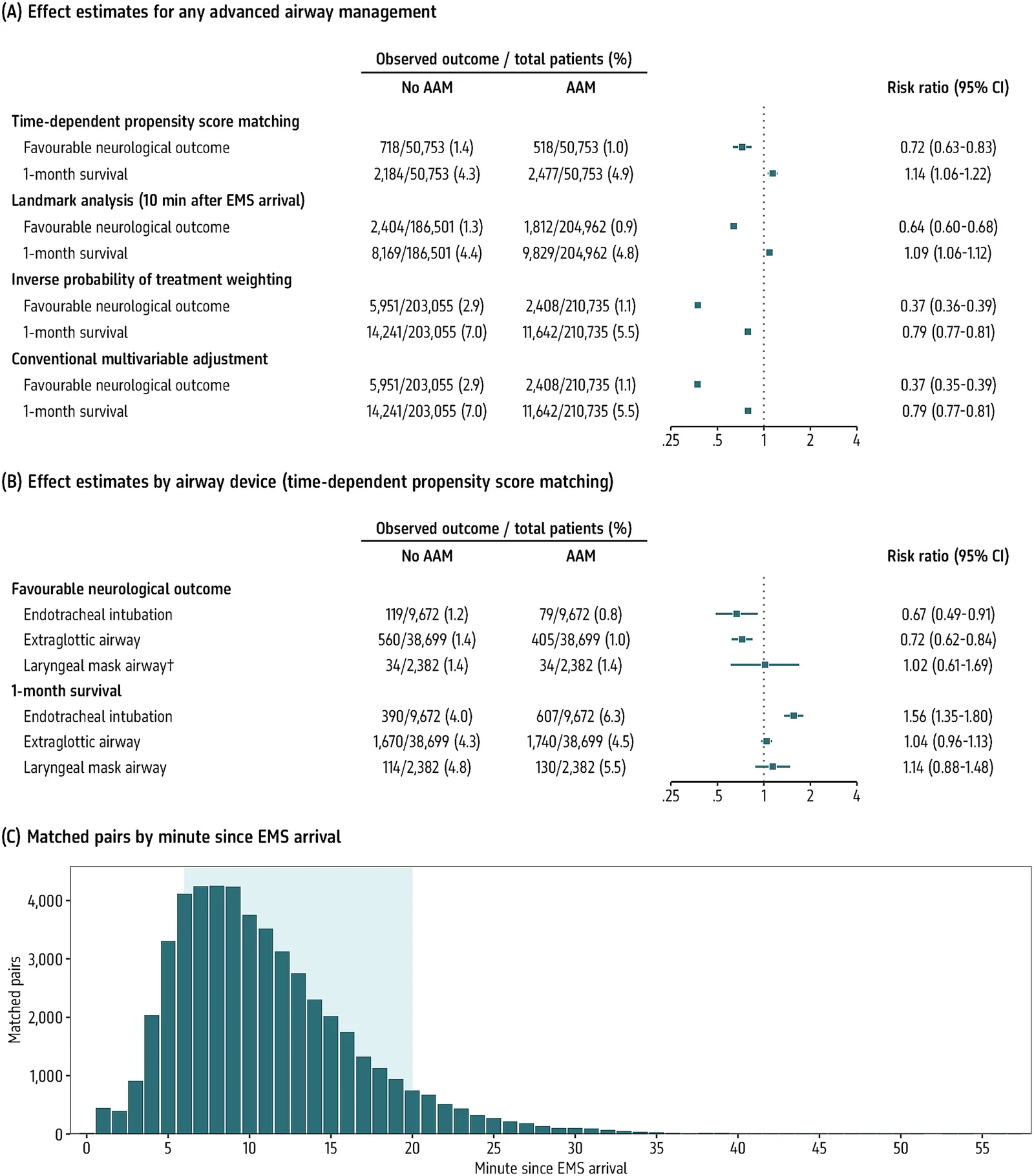
**Association of prehospital advanced airway management with clinical outcomes, by analytical approach and by airway device**. Forest plots showing the adjusted risk ratios (RRs) with 95% confidence intervals for the association between prehospital advanced airway management (AAM) and 1-month outcomes. Observed outcome rates (unadjusted counts/percentages) are shown for the AAM and no-AAM groups. (A) Any AAM versus none, under four analytical approaches. (B) Each airway device under time-dependent propensity score matching. (C) Histogram showing the distribution of matched pairs by the minute since emergency medical services arrival at which the airway was placed; the shaded area highlights the 6-to-20-min interval, where 79.3% of matches occurred. ^†^Fewer than 50 events in the exposed group; the confidence interval should not be relied on.

### Sensitivity analyses

Analyses restricted to complete cases were consistent with those from the imputed data, in both the prognostic models (Tables S9 and S10) and the intervention analyses (Table S12). Most associations were also stable across the four calendar periods, the clearest exception being prehospital ROSC, whose predictive strength declined over time (Tables S13 and S14).

## Discussion

In this nationwide PEA cohort, a witnessed arrest, shorter prehospital intervals, and prehospital ROSC were associated with a favourable neurological outcome in both cardiac and non-cardiac PEA. Other predictors differed. Younger age carried a greater advantage in cardiac PEA, and conversion to a shockable rhythm predicted a favourable outcome there but a worse one in non-cardiac PEA. Among survivors, a favourable neurological outcome was far more frequent in cardiac PEA. Previous studies have often been limited to smaller, regional cohorts or have incorporated in-hospital variables, such as comorbidities or clinical findings upon hospital arrival, which are unavailable to emergency clinicians during initial resuscitation.^8^, ^16^, ^17^ Adherence to the Utstein template makes our findings comparable with other EMS systems.^18^

The factors that behaved alike in the two aetiologies align with previous literature. A witnessed arrest, a well-documented predictor in OHCA populations,^17^ is a surrogate for a shorter time from collapse to emergency response. Both a longer response time and a longer post-arrival interval were associated with worse outcomes, to a similar degree in both aetiologies.^19^ Because both intervals begin at the emergency call, they omit the preceding no-flow period, so these associations probably understate the effect of elapsed time.

These predictors did not, however, operate uniformly. Conversion from a non-shockable to a shockable rhythm is a well-established positive prognostic factor,^20^, ^21^ but in our data it predicted a favourable neurological outcome only in cardiac PEA and a worse outcome in non-cardiac PEA. In cardiac PEA, the heart is the cause of the arrest, so conversion marks myocardium still able to fibrillate rather than to remain in PEA, and often a process, such as ischaemia, amenable to targeted therapies. In non-cardiac PEA, the cause lies outside the heart, so conversion carries little information about it, and its association with a worse outcome may reflect a prolonged resuscitation in a condition that it cannot reverse. Age, sex and prehospital ROSC also differed by aetiology. The advantage of younger age was greater in cardiac PEA. Younger age likely reflects greater physiological reserve and fewer comorbidities, enabling better tolerance to prolonged periods of low blood flow. Sex behaved in the same way. Male sex predicted a favourable outcome only in cardiac PEA, which is consistent with the sex difference in OHCA outcome being tied to the underlying cause rather than to sex itself. Prehospital ROSC was the strongest predictor in both groups, but a restored circulation was followed by neurological recovery more often in cardiac PEA. Taken together, these differences suggest that in cardiac PEA the arrest is more often the proximate event in a patient who can still recover, whereas in non-cardiac PEA the ceiling is set largely by the underlying cause. The two outcomes ranked the aetiologies in opposite directions. Cardiac PEA had the lower survival but a much higher proportion of neurologically intact survivors. Circulation is more often restored when the heart is not the failing organ, yet the insult has by then often injured the brain. In cardiac PEA, the failing organ is the one mechanical support replaces, and its cause is often treatable. Advanced therapies target neurological recovery rather than survival alone, so cardiac PEA may be the more promising group for them.

Bystander CPR is an established determinant of survival in OHCA overall,^22^ but in this cohort it showed little association with outcome. Restricting the cohort to PEA is part of the explanation. Because bystander CPR influences which rhythm is first recorded, conditioning on that rhythm introduces index-event selection bias, so the estimate within this stratum does not measure what whole-population estimates measure. The registry records only whether bystander CPR was given, not its quality or duration. PEA also encompasses states of profound cardiogenic shock where some cardiac output persists (pseudo-PEA), where the arrest onset is ambiguous, and asynchronous chest compressions could paradoxically impair cardiac output.^23^ These estimates should therefore not be read as questioning the public health value of bystander CPR.

Observational comparisons of prehospital interventions are biased from the outset, because untreated groups often include patients with early ROSC and therefore appear prognostically favourable.^24^, ^25^ Our conventional analysis showed apparent harm from both epinephrine and AAM. Once the timing of treatment was respected, both were associated with higher 1-month survival without a corresponding improvement in neurological outcome. For epinephrine, this mirrors the PARAMEDIC2 randomised trial,^26^ and for AAM, it matches a nationwide Japanese analysis of non-shockable arrests that used the same design.^27^ The neurological estimate for AAM, however, remained below the null, which most likely reflects confounding by indication, since airway management is undertaken preferentially in refractory arrests. Most matched treatments occurred within 20 min of EMS arrival, so the estimates apply mainly to that window. After accounting for these biases, the analyses establish neither harm nor benefit, so whether either intervention was performed has limited prognostic value.

Outcomes in PEA improved over most of the study period, consistent with progressive strengthening of the “chain of survival” in Japan.^28^, ^29^ The prognostic weight of most predictors was stable throughout, but the association between prehospital ROSC and a favourable outcome attenuated. As prehospital interventions became more widely used, more patients achieved ROSC, so transient ROSC in patients with little prospect of recovery may have become more common. Beyond these system-level changes, our findings have important implications for patient-level triage to advanced therapies. Ischaemic heart disease is implicated in up to 70% of cardiac PEA cases, and patients with pre-existing left ventricular dysfunction who experience acute coronary occlusion often present with PEA due to acute pump failure before transitioning to ventricular fibrillation.^9^, ^10^ This pathophysiology suggests that PEA due to myocardial infarction or heart failure may represent an early phase of arrest before widespread organ injury becomes irreversible. Such patients may be salvageable with a combination of prehospital interventions, cause-specific treatments, and mechanical circulatory support. However, PEA has traditionally been a relative contraindication for ECPR, so part of the poor-outcome group in our cohort may have been salvaged with ECPR. As recent evidence and guidelines now support ECPR for select patients with suspected reversible causes, our results identify prehospital predictors that could inform such decisions in future.^30^, ^31^

This study has several limitations. First, as an observational study, it remains subject to residual confounding that no adjustment can fully remove. This limitation is particularly relevant for both intervention analyses, and above all for AAM, whose status is to a large extent a surrogate of illness severity. The post-arrival interval raises a separate problem, reverse causation, in a system without field termination of resuscitation. Second, a cardiac origin is presumed by exclusion, so misclassification is unavoidable and the comparison between the groups is not a causal effect of aetiology. The non-cardiac group is more heterogeneous, and its composition could not be examined because the registry's subtype codes changed during the study period (Table S15). Third, we observed missing data for some key variables, such as conversion to a shockable rhythm. Although we addressed this using multiple imputation, which assumes only that data are missing at random, the complete-case analysis requires the stronger assumption of missingness completely at random; the two sets of estimates were consistent. Finally, our cohort predates the wider use of ECPR in PEA, so some patients with a poor predicted prognosis may not have been unsalvageable but simply untreated. The prognostic weight of these factors may therefore change as ECPR becomes standard.

## Conclusion

This nationwide analysis of PEA identified a witnessed arrest, shorter prehospital intervals, and prehospital ROSC as predictors of a favourable outcome in both cardiac and non-cardiac PEA; in cardiac PEA, younger age carried a far greater advantage and conversion to a shockable rhythm predicted a favourable outcome only there. Neurologically intact survival was more likely in cardiac PEA, although survival alone was not. The apparent harm from epinephrine and AAM was largely attenuated after accounting for methodological biases. These are independent prehospital predictors that could inform future risk stratification.

## Declaration of generative AI and AI-assisted technologies in the manuscript preparation process

During the preparation of this work, the author(s) used Claude by Anthropic to assist with English-language editing of the manuscript, including proofreading for grammar and consistency, and improving the clarity and phrasing of selected sentences. The author(s) reviewed and edited the output as needed and take full responsibility for the content of the published article.

## CRediT authorship contribution statement

**Shumpei Kosugi:** Writing – original draft, Project administration, Methodology, Formal analysis, Data curation, Conceptualization. **Yasunori Ueda:** Writing – review & editing, Supervision. **Haruhiko Abe:** Writing – review & editing. **Taro Takeuchi:** Writing – review & editing. **Koichi Inoue:** Writing – review & editing. **Yasushi Matsumura:** Writing – review & editing. **Naohiro Yonemoto:** Writing – review & editing, Validation, Supervision, Data curation. **Tetsuya Matoba:** Writing – review & editing, Validation, Supervision. **Yoshio Tahara:** Writing – review & editing, Validation, Supervision.

## Funding

This study received no funding.

## Declaration of competing interest

The authors declare that they have no known competing financial interests or personal relationships that could have appeared to influence the work reported in this paper.

## Figures and Tables

**Supplementary Fig. S1:**
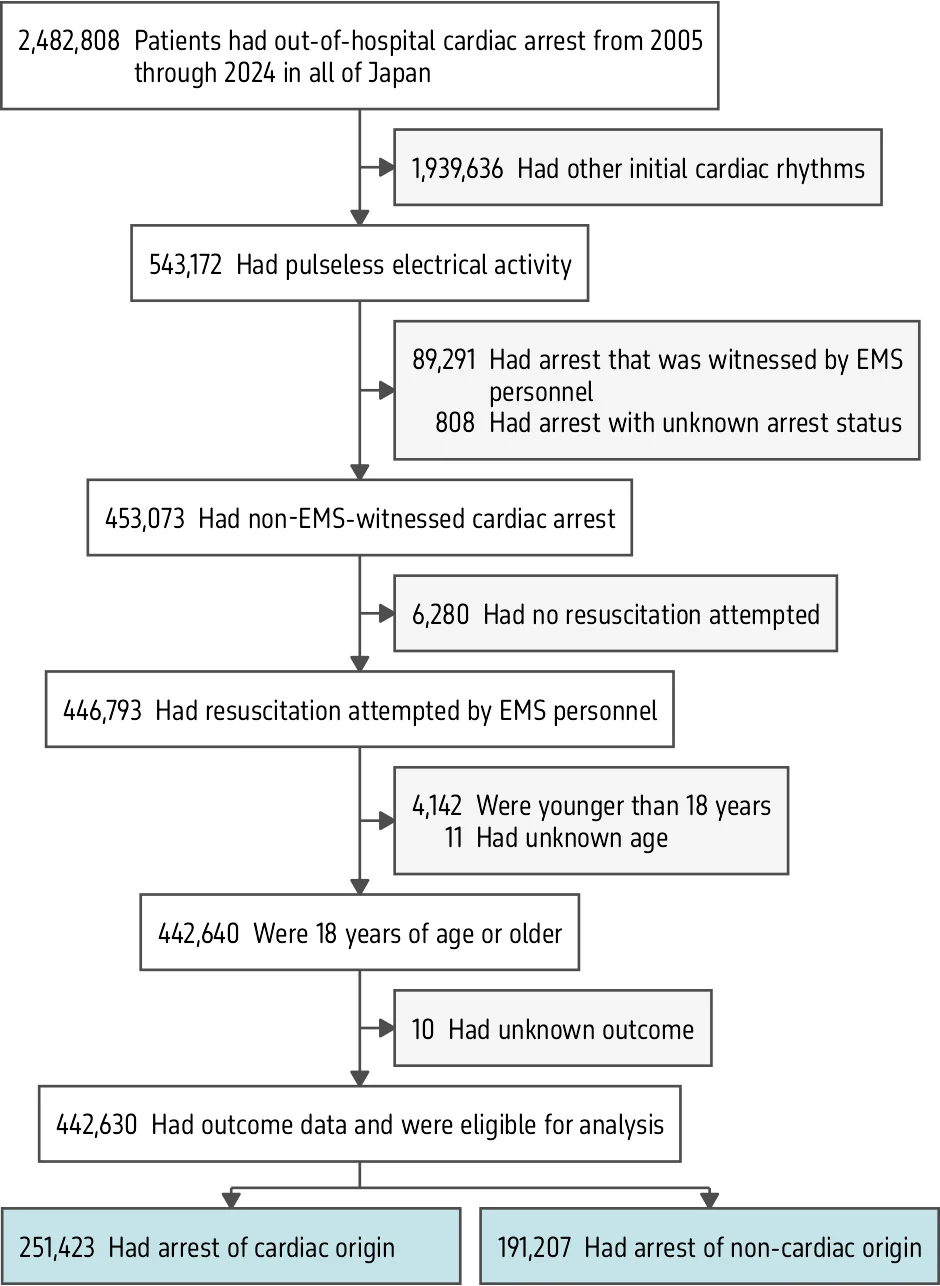
**Flow diagram of the study population.** The final two boxes show the division of the analysed cohort by presumed aetiology. *EMS, emergency medical services*.

**Supplementary Fig. S2:**
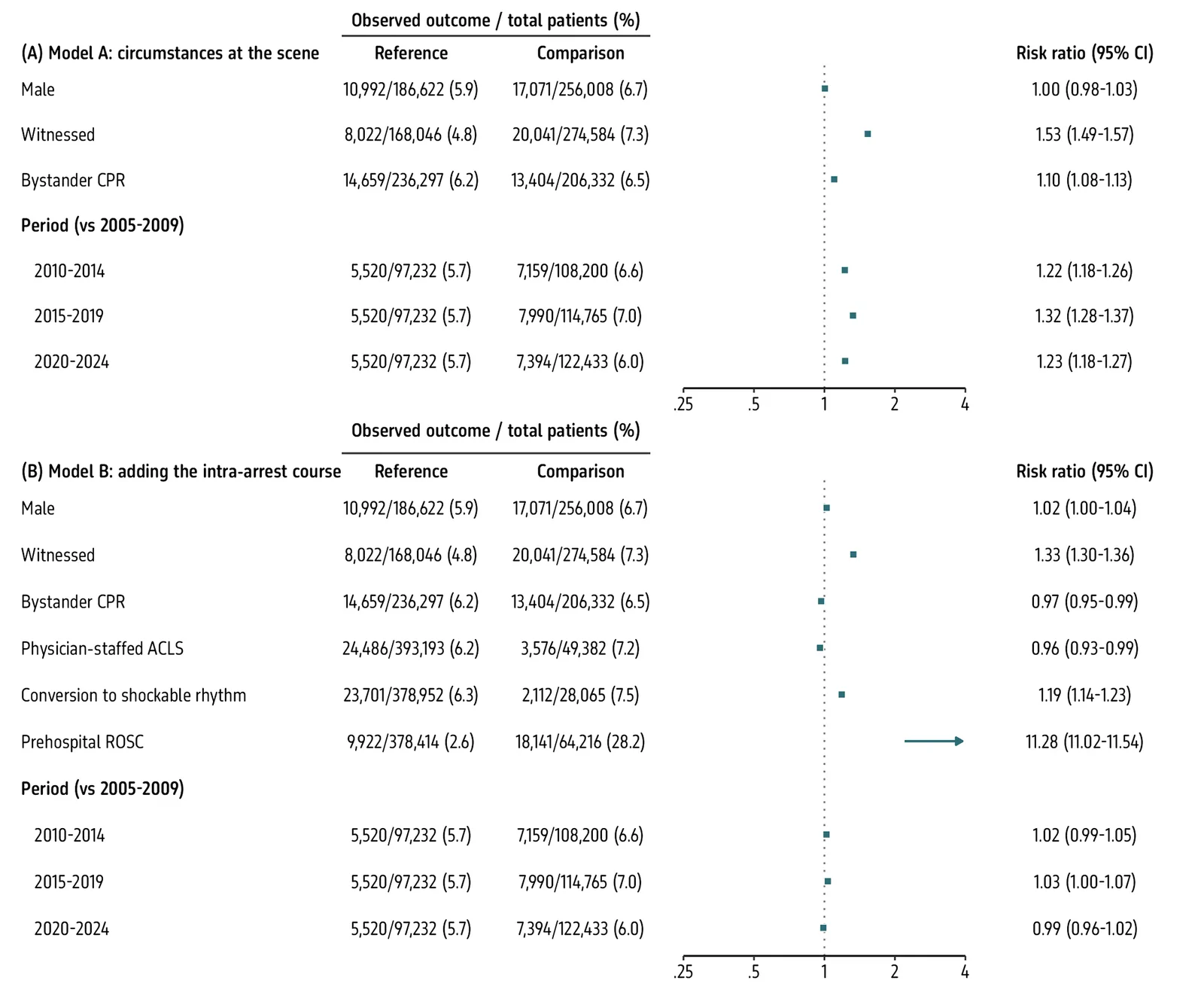
**Observed 1-month survival rates and adjusted risk ratios associated with prehospital factors.** Observed rates of 1-month survival (unadjusted counts/percentages) and adjusted risk ratios (RRs) with 95% confidence intervals for each prehospital factor, from (A) Model A and (B) Model B. Estimates outside the plotted range are shown as arrows. *ACLS, advanced cardiovascular life support; CPR, cardiopulmonary resuscitation; ROSC, return of spontaneous circulation*.

**Supplementary Fig. S3:**
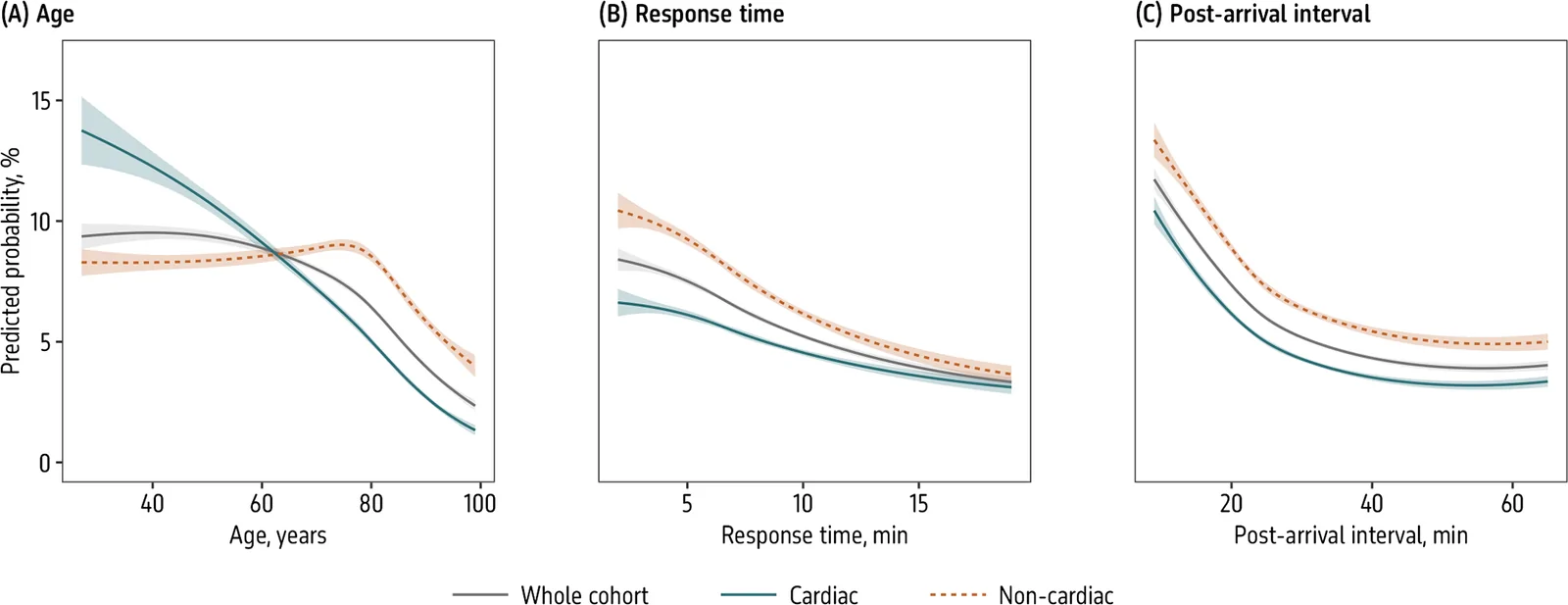
**Spline-based adjusted probabilities of 1-month survival by age and prehospital time.** Predicted probabilities of 1-month survival according to (A) age, (B) response time, and (C) the post-arrival interval, modelled using restricted cubic splines. The solid lines represent the adjusted probabilities, and the shaded areas represent the 95% confidence intervals. Each panel shows the whole cohort and the same variable estimated separately among patients with a cardiac and a non-cardiac aetiology.

**Supplementary Fig. S4:**
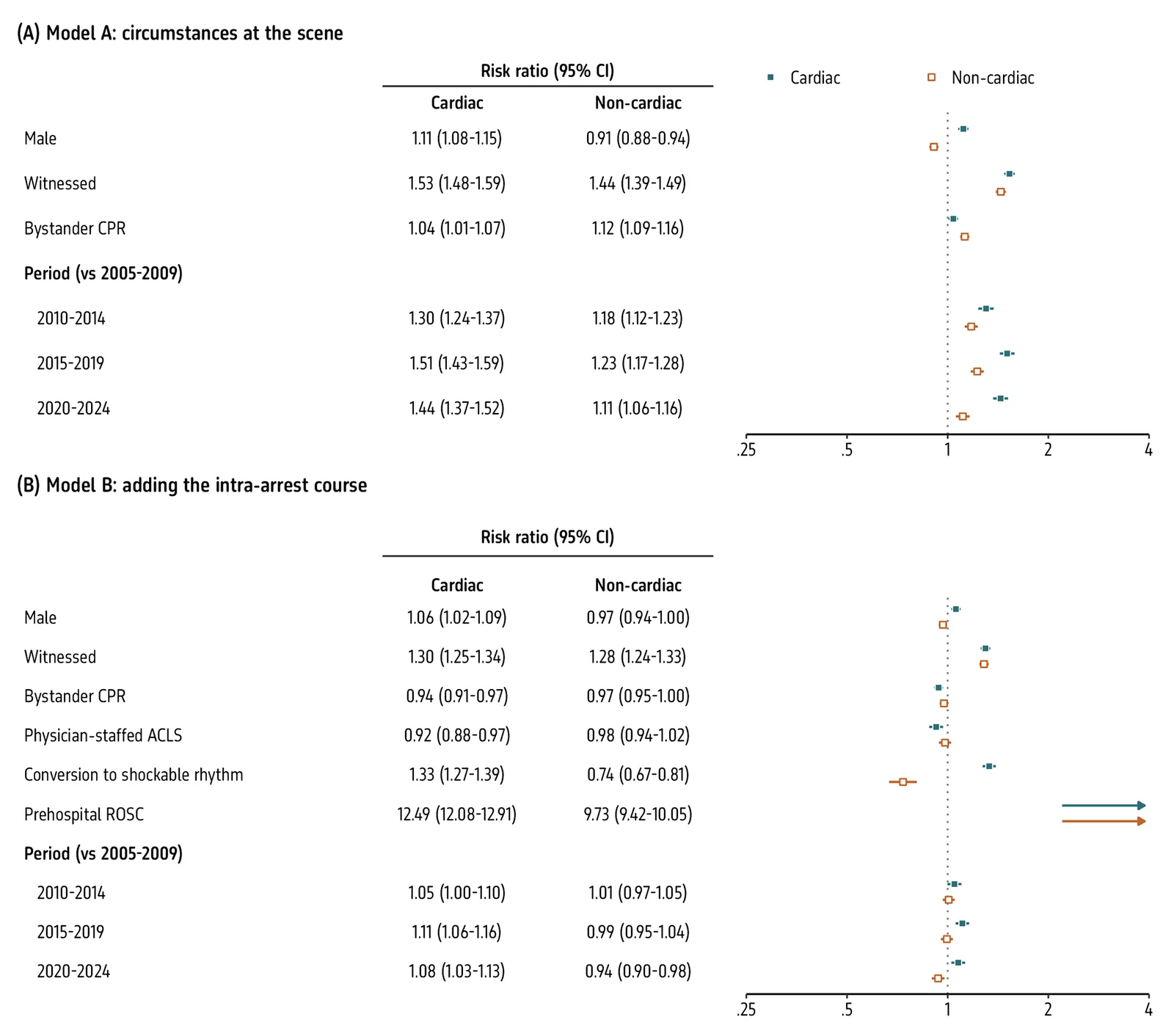
**Adjusted risk ratios for 1-month survival, by presumed aetiology.** Risk ratios (RRs) with 95% confidence intervals for each prehospital factor, estimated separately among patients with a cardiac and a non-cardiac aetiology, from (A) Model A and (B) Model B. Filled squares are cardiac and open squares are non-cardiac arrests. Estimates outside the plotted range are shown as arrows. *ACLS, advanced cardiovascular life support; CPR, cardiopulmonary resuscitation; ROSC, return of spontaneous circulation*.
