# Supplementary material for: Prehospital predictors of clinical outcomes in out-of-hospital cardiac arrest with pulseless electrical activity: a JCS-ReSS report
Source: Resusc Plus. 2026 Sep 28;32:101508. doi: 10.1016/j.resplu.2026.101508 (PMC13676978; doi:10.1016/j.resplu.2026.101508)
Supplement: Supplementary file 1 [file mmc10.docx]

**Table S6. Covariate balance for advanced airway management before and after time-dependent propensity score matching**

|  | Any AAM | | Endotracheal intubation | | Extraglottic airway | | Laryngeal mask airway | |
| --- | --- | --- | --- | --- | --- | --- | --- | --- |
| Covariate | Before | After | Before | After | Before | After | Before | After |
| Age | 0.127 | 0.060 | 0.132 | 0.049 | 0.123 | 0.066 | 0.168 | 0.051 |
| Male | 0.021 | 0.001 | 0.006 | 0.002 | 0.026 | <0.001 | 0.040 | <0.001 |
| Witnessed | 0.045 | 0.015 | 0.103 | 0.023 | 0.031 | 0.017 | 0.052 | 0.005 |
| Bystander CPR | 0.045 | 0.003 | 0.086 | 0.003 | 0.030 | 0.001 | 0.127 | 0.018 |
| Response time | 0.038 | 0.045 | 0.022 | 0.055 | 0.045 | 0.044 | 0.056 | 0.022 |
| Physician-staffed ACLS | 0.016 | 0.080 | 0.091 | 0.073 | 0.041 | 0.071 | 0.073 | 0.068 |
| Conversion to shockable rhythm | 0.112 | 0.082 | 0.105 | 0.080 | 0.113 | 0.082 | 0.112 | 0.100 |
| Non-cardiac aetiology | 0.082 | 0.006 | 0.120 | 0.012 | 0.134 | 0.004 | 0.069 | 0.013 |
| Calendar year | 0.105 | 0.013 | 0.098 | 0.020 | 0.112 | 0.014 | 0.158 | 0.024 |
| Values are absolute standardised mean differences between treated and control person-minutes, pooled over 30 imputed datasets. | | | | | | | | |
| Restricted to 2021-2024, the years in which the time of airway placement is recorded; calendar year is contrasted against 2021. | | | | | | | | |
| For covariates represented by several columns (Age, 4 columns; Response time, 4 columns; Calendar year, 3 columns), the largest absolute difference across those columns is shown. | | | | | | | | |
| ACLS, advanced cardiovascular life support; CPR, cardiopulmonary resuscitation; SMD, standardised mean difference. | | | | | | | | |
