# Supplementary material for: Prehospital predictors of clinical outcomes in out-of-hospital cardiac arrest with pulseless electrical activity: a JCS-ReSS report
Source: Resusc Plus. 2026 Sep 28;32:101508. doi: 10.1016/j.resplu.2026.101508 (PMC13676978; doi:10.1016/j.resplu.2026.101508)
Supplement: Supplementary file 2 [file mmc11.docx]

**Table S7. Covariate balance for advanced airway management before and after stabilised inverse probability of treatment weighting**

|  | Any AAM | | Endotracheal intubation | | Extraglottic airway | | Laryngeal mask airway | |
| --- | --- | --- | --- | --- | --- | --- | --- | --- |
| Covariate | Before | After | Before | After | Before | After | Before | After |
| Age | 0.107 | 0.001 | 0.125 | 0.015 | 0.104 | 0.001 | 0.133 | 0.022 |
| Male | 0.030 | <0.001 | 0.004 | 0.002 | 0.037 | <0.001 | 0.044 | 0.005 |
| Witnessed | 0.047 | <0.001 | 0.137 | 0.003 | 0.034 | <0.001 | 0.031 | 0.006 |
| Bystander CPR | 0.069 | <0.001 | 0.132 | 0.001 | 0.065 | 0.001 | 0.029 | 0.014 |
| Response time | 0.071 | <0.001 | 0.069 | 0.001 | 0.094 | 0.001 | 0.104 | 0.005 |
| Physician-staffed ACLS | 0.056 | <0.001 | 0.041 | 0.001 | 0.083 | 0.001 | 0.059 | 0.060 |
| Conversion to shockable rhythm | 0.097 | <0.001 | 0.086 | <0.001 | 0.100 | <0.001 | 0.099 | 0.006 |
| Non-cardiac aetiology | 0.104 | <0.001 | 0.104 | 0.022 | 0.152 | 0.001 | 0.165 | 0.015 |
| Calendar period | 0.143 | <0.001 | 0.144 | 0.003 | 0.178 | <0.001 | 0.254 | 0.008 |
| Values are absolute standardised mean differences, pooled over 30 imputed datasets; Before, unweighted; After, after stabilised inverse probability of treatment weighting. | | | | | | | | |
| For covariates represented by several columns (Age, 4 columns; Response time, 4 columns; Calendar period, 3 columns), the largest absolute difference across those columns is shown. | | | | | | | | |
| ACLS, advanced cardiovascular life support; CPR, cardiopulmonary resuscitation; SMD, standardised mean difference. | | | | | | | | |
