# Supplementary material for: Prehospital predictors of clinical outcomes in out-of-hospital cardiac arrest with pulseless electrical activity: a JCS-ReSS report
Source: Resusc Plus. 2026 Sep 28;32:101508. doi: 10.1016/j.resplu.2026.101508 (PMC13676978; doi:10.1016/j.resplu.2026.101508)
Supplement: Supplementary file 3 [file mmc12.docx]

**Table S8. Baseline characteristics of the study cohort, by study period**

|  | Study period | | | | |  |
| --- | --- | --- | --- | --- | --- | --- |
| Variable | Overall (n = 442,630) | 2005-2009 (n = 97,232) | 2010-2014 (n = 108,200) | 2015-2019 (n = 114,765) | 2020-2024 (n = 122,433) | Missing n (%) |
| **Patient characteristics** |  |  |  |  |  |  |
| Age, years | 80 [70, 87] | 78 [67, 85] | 79 [69, 86] | 80 [70, 87] | 81 [72, 88] | 0 (0.0) |
| Male | 256,008 (57.8) | 56,491 (58.1) | 61,808 (57.1) | 65,795 (57.3) | 71,914 (58.7) | 0 (0.0) |
| **Arrest circumstances** |  |  |  |  |  |  |
| Witnessed by bystander | 274,584 (62.0) | 56,113 (57.7) | 67,677 (62.5) | 73,342 (63.9) | 77,452 (63.3) | 0 (0.0) |
| Bystander CPR | 206,332 (46.6) | 37,182 (38.2) | 48,491 (44.8) | 57,233 (49.9) | 63,426 (51.8) | 1 (0.0) |
| **Time intervals, min** |  |  |  |  |  |  |
| Response time (call to scene arrival) | 7 [6, 9] | 6 [5, 8] | 7 [5, 9] | 7 [6, 9] | 8 [6, 10] | 287 (0.1) |
| Post-arrival interval (scene arrival to hospital arrival) | 24 [19, 31] | 22 [17, 29] | 24 [18, 31] | 24 [19, 31] | 25 [20, 32] | 565 (0.1) |
| No-flow time (collapse to first CPR)† | 5 [1, 11] | 6 [2, 11] | 6 [1, 11] | 5 [1, 10] | 5 [1, 11] | 1,110 (0.4) |
| **Presumed aetiology** |  |  |  |  |  |  |
| Cardiac origin | 251,423 (56.8) | 51,310 (52.8) | 58,957 (54.5) | 66,495 (57.9) | 74,661 (61.0) | 0 (0.0) |
| Non-cardiac origin | 191,207 (43.2) | 45,922 (47.2) | 49,243 (45.5) | 48,270 (42.1) | 47,772 (39.0) | 0 (0.0) |
| **Resuscitation course** |  |  |  |  |  |  |
| Conversion to shockable rhythm | 28,065 (6.9) | 5,893 (6.1) | 6,643 (6.1) | 7,309 (7.9) | 8,220 (7.6) | 35,613 (8.0) |
| Physician-staffed ACLS | 49,382 (11.2) | 15,277 (15.7) | 11,113 (10.3) | 11,292 (9.8) | 11,700 (9.6) | 55 (0.0) |
| Prehospital epinephrine | 122,638 (29.3) | 7,752 (8.0) | 25,976 (24.0) | 38,062 (38.6) | 50,848 (44.6) | 24,647 (5.6) |
| Advanced airway management: none | 203,193 (49.1) | 49,649 (51.1) | 59,577 (55.1) | 45,968 (47.0) | 47,999 (43.3) | 28,566 (6.5) |
| Endotracheal intubation | 39,242 (9.5) | 7,185 (7.4) | 9,641 (8.9) | 10,650 (10.9) | 11,766 (10.6) | 28,566 (6.5) |
| Extraglottic airway | 152,598 (36.9) | 30,507 (31.4) | 35,448 (32.8) | 38,521 (39.4) | 48,122 (43.4) | 28,566 (6.5) |
| Laryngeal mask airway | 19,031 (4.6) | 9,891 (10.2) | 3,534 (3.3) | 2,616 (2.7) | 2,990 (2.7) | 28,566 (6.5) |
| Prehospital ROSC | 64,216 (14.5) | 9,439 (9.7) | 15,940 (14.7) | 19,034 (16.6) | 19,803 (16.2) | 0 (0.0) |
| **Outcomes** |  |  |  |  |  |  |
| Favourable neurological outcome at 1 month | 9,348 (2.1) | 1,512 (1.6) | 2,270 (2.1) | 2,905 (2.5) | 2,661 (2.2) | 0 (0.0) |
| Survival at 1 month | 28,063 (6.3) | 5,520 (5.7) | 7,159 (6.6) | 7,990 (7.0) | 7,394 (6.0) | 0 (0.0) |
| Data are median [interquartile range] or n (%); percentages are calculated among cases with non-missing data, and the Missing column refers to the whole cohort. | | | | | | |
| † Bystander-witnessed arrests only (n = 274,584). | | | | | | |
| ACLS, advanced cardiovascular life support; CPR, cardiopulmonary resuscitation; ROSC, return of spontaneous circulation. | | | | | | |
