# Supplementary material for: Prehospital predictors of clinical outcomes in out-of-hospital cardiac arrest with pulseless electrical activity: a JCS-ReSS report
Source: Resusc Plus. 2026 Sep 28;32:101508. doi: 10.1016/j.resplu.2026.101508 (PMC13676978; doi:10.1016/j.resplu.2026.101508)
Supplement: Supplementary file 4 [file mmc13.docx]

**Table S9. Prehospital predictors of a favourable neurological outcome: risk ratios from Model A and Model B, estimated after multiple imputation and among complete cases**

|  | Model A (at the scene) | | Model B (at hospital arrival) | |
| --- | --- | --- | --- | --- |
| Variable | Multiple imputation RR (95% CI) | Complete cases RR (95% CI) | Multiple imputation RR (95% CI) | Complete cases RR (95% CI) |
| Age, 80 vs 60 years | 0.50 (0.47 to 0.53) | 0.50 (0.47 to 0.53) | 0.56 (0.53 to 0.59) | 0.56 (0.53 to 0.59) |
| Male | 1.12 (1.08 to 1.17) | 1.13 (1.08 to 1.18) | 1.15 (1.10 to 1.19) | 1.14 (1.09 to 1.19) |
| Witnessed | 1.65 (1.58 to 1.73) | 1.65 (1.58 to 1.73) | 1.39 (1.33 to 1.45) | 1.40 (1.33 to 1.46) |
| Bystander CPR | 0.98 (0.94 to 1.02) | 0.98 (0.94 to 1.02) | 0.82 (0.79 to 0.85) | 0.82 (0.79 to 0.86) |
| Response time, 10 vs 5 min | 0.66 (0.63 to 0.70) | 0.66 (0.63 to 0.70) | 0.79 (0.75 to 0.82) | 0.80 (0.76 to 0.84) |
| Physician-staffed ACLS | — | — | 0.86 (0.81 to 0.92) | 0.87 (0.82 to 0.93) |
| Conversion to shockable rhythm | — | — | 1.51 (1.41 to 1.61) | 1.51 (1.42 to 1.61) |
| Prehospital ROSC | — | — | 23.31 (22.18 to 24.50) | 23.32 (22.14 to 24.56) |
| Post-arrival interval, 30 vs 20 min | — | — | 0.67 (0.64 to 0.69) | 0.66 (0.63 to 0.69) |
| **Calendar period (vs 2005-2009)** |  |  |  |  |
| 2010-2014 | 1.46 (1.37 to 1.56) | 1.46 (1.37 to 1.56) | 1.15 (1.08 to 1.23) | 1.15 (1.08 to 1.22) |
| 2015-2019 | 1.86 (1.75 to 1.98) | 1.85 (1.74 to 1.97) | 1.34 (1.26 to 1.42) | 1.35 (1.27 to 1.44) |
| 2020-2024 | 1.74 (1.63 to 1.85) | 1.73 (1.63 to 1.85) | 1.31 (1.23 to 1.39) | 1.32 (1.24 to 1.41) |
| Values are risk ratios (95% confidence intervals); — indicates that the variable is not in that model. | | | | |
| Complete-case analyses include 442,342 patients for Model A and 406,216 for Model B. | | | | |
| ACLS, advanced cardiovascular life support; CI, confidence interval; CPR, cardiopulmonary resuscitation; RR, risk ratio; ROSC, return of spontaneous circulation. | | | | |
