# Supplementary material for: Prehospital predictors of clinical outcomes in out-of-hospital cardiac arrest with pulseless electrical activity: a JCS-ReSS report
Source: Resusc Plus. 2026 Sep 28;32:101508. doi: 10.1016/j.resplu.2026.101508 (PMC13676978; doi:10.1016/j.resplu.2026.101508)
Supplement: Supplementary file 5 [file mmc14.docx]

**Table S10. Prehospital predictors of 1-month survival: risk ratios from Model A and Model B, estimated after multiple imputation and among complete cases**

|  | Model A (at the scene) | | Model B (at hospital arrival) | |
| --- | --- | --- | --- | --- |
| Variable | Multiple imputation RR (95% CI) | Complete cases RR (95% CI) | Multiple imputation RR (95% CI) | Complete cases RR (95% CI) |
| Age, 80 vs 60 years | 0.72 (0.70 to 0.75) | 0.72 (0.70 to 0.75) | 0.79 (0.77 to 0.82) | 0.80 (0.77 to 0.82) |
| Male | 1.00 (0.98 to 1.03) | 1.00 (0.98 to 1.03) | 1.02 (1.00 to 1.04) | 1.02 (1.00 to 1.05) |
| Witnessed | 1.53 (1.49 to 1.57) | 1.53 (1.49 to 1.57) | 1.33 (1.30 to 1.36) | 1.32 (1.29 to 1.36) |
| Bystander CPR | 1.10 (1.08 to 1.13) | 1.10 (1.08 to 1.13) | 0.97 (0.95 to 0.99) | 0.98 (0.95 to 1.00) |
| Response time, 10 vs 5 min | 0.70 (0.68 to 0.72) | 0.70 (0.68 to 0.72) | 0.80 (0.78 to 0.83) | 0.81 (0.79 to 0.83) |
| Physician-staffed ACLS | — | — | 0.96 (0.93 to 0.99) | 0.96 (0.93 to 1.00) |
| Conversion to shockable rhythm | — | — | 1.19 (1.14 to 1.23) | 1.19 (1.14 to 1.24) |
| Prehospital ROSC | — | — | 11.28 (11.02 to 11.54) | 11.20 (10.93 to 11.47) |
| Post-arrival interval, 30 vs 20 min | — | — | 0.71 (0.69 to 0.72) | 0.70 (0.69 to 0.72) |
| **Calendar period (vs 2005-2009)** |  |  |  |  |
| 2010-2014 | 1.22 (1.18 to 1.26) | 1.22 (1.18 to 1.26) | 1.02 (0.99 to 1.05) | 1.02 (0.99 to 1.05) |
| 2015-2019 | 1.32 (1.28 to 1.37) | 1.32 (1.28 to 1.37) | 1.03 (1.00 to 1.07) | 1.04 (1.01 to 1.08) |
| 2020-2024 | 1.23 (1.18 to 1.27) | 1.22 (1.18 to 1.27) | 0.99 (0.96 to 1.02) | 0.99 (0.96 to 1.02) |
| Values are risk ratios (95% confidence intervals); — indicates that the variable is not in that model. | | | | |
| Complete-case analyses include 442,342 patients for Model A and 406,216 for Model B. | | | | |
| ACLS, advanced cardiovascular life support; CI, confidence interval; CPR, cardiopulmonary resuscitation; RR, risk ratio; ROSC, return of spontaneous circulation. | | | | |
