# Supplementary material for: Prehospital predictors of clinical outcomes in out-of-hospital cardiac arrest with pulseless electrical activity: a JCS-ReSS report
Source: Resusc Plus. 2026 Sep 28;32:101508. doi: 10.1016/j.resplu.2026.101508 (PMC13676978; doi:10.1016/j.resplu.2026.101508)
Supplement: Supplementary file 6 [file mmc15.docx]

**Table S11. Effects of prehospital interventions estimated separately among patients with a cardiac and a non-cardiac aetiology**

| Analysis | Cardiac RR (95% CI) | Non-cardiac RR (95% CI) | Non-cardiac / cardiac ratio of RR (95% CI) | Non-cardiac minus cardiac difference in RD % points (95% CI) |
| --- | --- | --- | --- | --- |
| **Epinephrine** |  |  |  |  |
| Favourable neurological outcome | 0.99 (0.84 to 1.17) | 1.02 (0.83 to 1.25) | 1.03 (0.79 to 1.34) | 0.03 (-0.24 to 0.30) |
| 1-month survival | 1.39 (1.27 to 1.52) | 1.55 (1.41 to 1.69) | 1.11 (0.98 to 1.26) | 1.08 (0.56 to 1.60) |
| **Advanced airway management (any device)** |  |  |  |  |
| Favourable neurological outcome | 0.73 (0.61 to 0.86) | 0.69 (0.55 to 0.87) | 0.95 (0.72 to 1.27) | 0.02 (-0.34 to 0.37) |
| 1-month survival | 1.06 (0.96 to 1.17) | 1.22 (1.10 to 1.36) | 1.15 (1.00 to 1.33) | 0.90 (0.22 to 1.58) |
| **Endotracheal intubation** |  |  |  |  |
| Favourable neurological outcome† | 0.60 (0.40 to 0.91) | 0.77 (0.47 to 1.26) | 1.27 (0.67 to 2.44) | 0.27 (-0.40 to 0.95) |
| 1-month survival | 1.32 (1.06 to 1.65) | 1.77 (1.45 to 2.16) | 1.34 (0.99 to 1.80) | 2.51 (1.11 to 3.91) |
| **Extraglottic airway** |  |  |  |  |
| Favourable neurological outcome | 0.76 (0.63 to 0.92) | 0.63 (0.48 to 0.83) | 0.82 (0.59 to 1.15) | -0.16 (-0.59 to 0.27) |
| 1-month survival | 1.02 (0.91 to 1.13) | 1.07 (0.95 to 1.21) | 1.05 (0.90 to 1.24) | 0.30 (-0.47 to 1.07) |
| **Laryngeal mask airway** |  |  |  |  |
| Favourable neurological outcome† | 0.89 (0.48 to 1.68) | 1.31 (0.52 to 3.31) | 1.46 (0.48 to 4.48) | 0.50 (-1.00 to 2.00) |
| 1-month survival† | 1.35 (0.90 to 2.03) | 0.95 (0.67 to 1.36) | 0.70 (0.41 to 1.21) | -1.58 (-4.48 to 1.32) |
| Values are risk ratios (95% confidence intervals); the ratio divides the non-cardiac by the cardiac risk ratio, and the difference subtracts the cardiac from the non-cardiac risk difference. | | | | |
| † Estimate flagged as unstable in at least one aetiology: fewer than 50 events in the treated group, or a maximum absolute standardised mean difference above 0.10 after matching. | | | | |
| CI, confidence interval; RD, risk difference; RR, risk ratio. | | | | |
