# Supplementary material for: Prehospital predictors of clinical outcomes in out-of-hospital cardiac arrest with pulseless electrical activity: a JCS-ReSS report
Source: Resusc Plus. 2026 Sep 28;32:101508. doi: 10.1016/j.resplu.2026.101508 (PMC13676978; doi:10.1016/j.resplu.2026.101508)
Supplement: Supplementary file 7 [file mmc16.docx]

**Table S12. Effects of prehospital interventions estimated after multiple imputation and among complete cases**

|  |  | Favourable neurological outcome | | 1-month survival | |
| --- | --- | --- | --- | --- | --- |
| Analysis | Complete cases, n | Multiple imputation | Complete cases | Multiple imputation | Complete cases |
| **Epinephrine** |  |  |  |  |  |
| Time-dependent propensity score matching | 285,104 | 1.00 (0.88 to 1.14) | 1.02 (0.89 to 1.15) | 1.46 (1.37 to 1.56) | 1.46 (1.37 to 1.56) |
| **Advanced airway management (any device)** |  |  |  |  |  |
| Time-dependent propensity score matching | 86,220 | 0.72 (0.63 to 0.83) | 0.74 (0.64 to 0.84) | 1.14 (1.06 to 1.22) | 1.14 (1.07 to 1.23) |
| Landmark analysis (10 min after EMS arrival) | 374,659 | 0.64 (0.60 to 0.68) | 0.65 (0.61 to 0.69) | 1.09 (1.06 to 1.12) | 1.10 (1.07 to 1.13) |
| Inverse probability of treatment weighting | 396,494 | 0.37 (0.36 to 0.39) | 0.38 (0.36 to 0.40) | 0.79 (0.77 to 0.81) | 0.79 (0.78 to 0.81) |
| Values are risk ratios (95% confidence intervals). | | | | | |
| The number of complete cases differs between analyses because the time of the intervention is recorded only from 2011 for epinephrine and from 2021 for airway management. | | | | | |
| AAM, advanced airway management; CC, complete cases; CI, confidence interval; EMS, emergency medical services; MI, multiple imputation. | | | | | |
