# Supplementary material for: Prehospital predictors of clinical outcomes in out-of-hospital cardiac arrest with pulseless electrical activity: a JCS-ReSS report
Source: Resusc Plus. 2026 Sep 28;32:101508. doi: 10.1016/j.resplu.2026.101508 (PMC13676978; doi:10.1016/j.resplu.2026.101508)
Supplement: Supplementary file 8 [file mmc17.docx]

**Table S13. Prehospital predictors of clinical outcomes, estimated separately within each study period**

|  | Study period | | | |  |
| --- | --- | --- | --- | --- | --- |
| Variable | 2005-2009 RR (95% CI) | 2010-2014 RR (95% CI) | 2015-2019 RR (95% CI) | 2020-2024 RR (95% CI) | 2020-2024 versus 2005-2009 ratio of RR (95% CI) |
| **Favourable neurological outcome — Model A: circumstances at the scene** |  |  |  |  |  |
| Age, 80 vs 60 years | 0.51 (0.45-0.58) | 0.49 (0.44-0.55) | 0.50 (0.45-0.55) | 0.50 (0.45-0.55) | 0.97 (0.82-1.15) |
| Male | 1.02 (0.92-1.13) | 1.10 (1.01-1.20) | 1.14 (1.05-1.23) | 1.20 (1.11-1.31) | 1.18 (1.03-1.35) |
| Witnessed | 1.89 (1.69-2.11) | 1.75 (1.59-1.92) | 1.63 (1.50-1.76) | 1.48 (1.36-1.61) | 0.78 (0.68-0.90) |
| Bystander CPR | 0.88 (0.79-0.98) | 1.05 (0.97-1.14) | 0.96 (0.90-1.04) | 1.00 (0.92-1.08) | 1.14 (1.00-1.29) |
| Response time, 10 vs 5 min | 0.60 (0.52-0.69) | 0.64 (0.58-0.71) | 0.70 (0.64-0.77) | 0.67 (0.61-0.73) | 1.11 (0.93-1.32) |
| **Favourable neurological outcome — Model B: adding the intra-arrest course** |  |  |  |  |  |
| Age, 80 vs 60 years | 0.64 (0.56-0.72) | 0.57 (0.51-0.63) | 0.55 (0.50-0.61) | 0.53 (0.48-0.58) | 0.83 (0.71-0.97) |
| Male | 1.12 (1.01-1.23) | 1.14 (1.05-1.23) | 1.14 (1.06-1.23) | 1.19 (1.10-1.28) | 1.06 (0.93-1.20) |
| Witnessed | 1.44 (1.30-1.61) | 1.44 (1.31-1.57) | 1.37 (1.26-1.48) | 1.34 (1.24-1.45) | 0.93 (0.81-1.06) |
| Bystander CPR | 0.74 (0.67-0.82) | 0.88 (0.81-0.95) | 0.81 (0.76-0.87) | 0.83 (0.77-0.89) | 1.12 (0.99-1.27) |
| Response time, 10 vs 5 min | 0.74 (0.65-0.85) | 0.79 (0.71-0.87) | 0.80 (0.74-0.87) | 0.80 (0.73-0.87) | 1.08 (0.91-1.27) |
| Physician-staffed ACLS | 0.91 (0.80-1.03) | 0.88 (0.78-1.00) | 0.82 (0.73-0.91) | 0.89 (0.79-0.99) | 0.97 (0.82-1.16) |
| Conversion to shockable rhythm | 1.64 (1.40-1.91) | 1.55 (1.36-1.76) | 1.44 (1.28-1.62) | 1.48 (1.32-1.66) | 0.91 (0.75-1.10) |
| Prehospital ROSC | 33.39 (29.57-37.69) | 27.80 (25.00-30.91) | 21.26 (19.43-23.27) | 17.77 (16.25-19.42) | 0.53 (0.46-0.62) |
| Post-arrival interval, 30 vs 20 min | 0.78 (0.71-0.86) | 0.63 (0.58-0.68) | 0.69 (0.64-0.74) | 0.63 (0.58-0.68) | 0.81 (0.72-0.92) |
| **1-month survival — Model A: circumstances at the scene** |  |  |  |  |  |
| Age, 80 vs 60 years | 0.80 (0.75-0.86) | 0.72 (0.68-0.77) | 0.71 (0.67-0.76) | 0.68 (0.64-0.72) | 0.85 (0.77-0.93) |
| Male | 0.97 (0.92-1.03) | 0.98 (0.93-1.02) | 1.02 (0.98-1.07) | 1.04 (0.99-1.09) | 1.07 (0.99-1.15) |
| Witnessed | 1.59 (1.50-1.68) | 1.62 (1.54-1.71) | 1.54 (1.47-1.62) | 1.39 (1.32-1.46) | 0.88 (0.81-0.94) |
| Bystander CPR | 1.14 (1.08-1.20) | 1.12 (1.07-1.18) | 1.06 (1.01-1.10) | 1.10 (1.05-1.15) | 0.97 (0.90-1.03) |
| Response time, 10 vs 5 min | 0.64 (0.60-0.69) | 0.72 (0.68-0.76) | 0.72 (0.69-0.76) | 0.68 (0.64-0.72) | 1.06 (0.97-1.15) |
| **1-month survival — Model B: adding the intra-arrest course** |  |  |  |  |  |
| Age, 80 vs 60 years | 0.92 (0.86-0.98) | 0.81 (0.76-0.85) | 0.77 (0.73-0.82) | 0.71 (0.67-0.75) | 0.77 (0.71-0.84) |
| Male | 1.04 (0.99-1.09) | 1.01 (0.96-1.05) | 1.02 (0.98-1.07) | 1.03 (0.99-1.08) | 0.99 (0.93-1.06) |
| Witnessed | 1.32 (1.25-1.38) | 1.38 (1.32-1.45) | 1.33 (1.27-1.39) | 1.28 (1.22-1.34) | 0.97 (0.91-1.04) |
| Bystander CPR | 1.02 (0.97-1.07) | 0.99 (0.95-1.03) | 0.93 (0.89-0.97) | 0.95 (0.92-0.99) | 0.94 (0.88-1.00) |
| Response time, 10 vs 5 min | 0.76 (0.71-0.81) | 0.85 (0.80-0.89) | 0.81 (0.77-0.85) | 0.78 (0.74-0.83) | 1.03 (0.95-1.12) |
| Physician-staffed ACLS | 0.94 (0.88-1.01) | 1.00 (0.94-1.07) | 0.93 (0.88-0.99) | 0.96 (0.90-1.02) | 1.02 (0.93-1.12) |
| Conversion to shockable rhythm | 1.14 (1.04-1.25) | 1.11 (1.02-1.20) | 1.21 (1.12-1.30) | 1.24 (1.15-1.33) | 1.09 (0.97-1.22) |
| Prehospital ROSC | 12.17 (11.59-12.79) | 11.49 (10.97-12.03) | 11.55 (11.04-12.08) | 10.26 (9.80-10.73) | 0.84 (0.79-0.90) |
| Post-arrival interval, 30 vs 20 min | 0.78 (0.74-0.81) | 0.68 (0.65-0.71) | 0.72 (0.69-0.75) | 0.67 (0.64-0.70) | 0.87 (0.81-0.92) |

Values are risk ratios (95% confidence intervals). Each column is a separate model fitted within that period, not a term of an interaction model; the last column divides the risk ratio in 2020-2024 by that in 2005-2009.

ACLS, advanced cardiovascular life support; CI, confidence interval; CPR, cardiopulmonary resuscitation; RR, risk ratio; ROSC, return of spontaneous circulation.
