# Supplementary material for: Prehospital predictors of clinical outcomes in out-of-hospital cardiac arrest with pulseless electrical activity: a JCS-ReSS report
Source: Resusc Plus. 2026 Sep 28;32:101508. doi: 10.1016/j.resplu.2026.101508 (PMC13676978; doi:10.1016/j.resplu.2026.101508)
Supplement: Supplementary file 9 [file mmc18.docx]

**Table S14. Effects of prehospital interventions, estimated separately within each study period**

|  | Study period | | | |  |
| --- | --- | --- | --- | --- | --- |
| Analysis | 2005-2009 RR (95% CI) | 2010-2014 RR (95% CI) | 2015-2019 RR (95% CI) | 2020-2024 RR (95% CI) | Last versus first period available ratio of RR (95% CI) |
| **Epinephrine (time-dependent propensity score matching)** |  |  |  |  |  |
| Favourable neurological outcome |  | 1.08 (0.83-1.42) | 1.20 (0.98-1.46) | 0.85 (0.70-1.04) | 0.79 (0.56-1.10) |
| 1-month survival |  | 1.39 (1.22-1.57) | 1.60 (1.44-1.77) | 1.40 (1.26-1.55) | 1.01 (0.86-1.19) |
| **Advanced airway management (landmark analysis, 10 min after EMS arrival)** |  |  |  |  |  |
| Favourable neurological outcome | 0.75 (0.65-0.87) | 0.70 (0.62-0.79) | 0.68 (0.61-0.77) | 0.50 (0.45-0.56) | 0.67 (0.56-0.81) |
| 1-month survival | 1.13 (1.06-1.20) | 1.11 (1.05-1.17) | 1.08 (1.02-1.14) | 1.04 (0.98-1.10) | 0.92 (0.84-1.00) |
| Values are risk ratios (95% confidence intervals). Each column is a separate analysis restricted to that period; the last column divides the risk ratio in the last period available by that in the first. | | | | | |
| Cells are empty for 2005-2009 in the epinephrine analysis because the time of epinephrine administration was not recorded in those years. | | | | | |
| AAM, advanced airway management; CI, confidence interval; RR, risk ratio. | | | | | |
