# Supplementary material for: Prehospital predictors of clinical outcomes in out-of-hospital cardiac arrest with pulseless electrical activity: a JCS-ReSS report
Source: Resusc Plus. 2026 Sep 28;32:101508. doi: 10.1016/j.resplu.2026.101508 (PMC13676978; doi:10.1016/j.resplu.2026.101508)
Supplement: Supplementary file 10 [file mmc19.docx]

**Table S15. Recorded subtype codes for arrests of non-cardiac origin, by coding era**

|  | Coding era | | |
| --- | --- | --- | --- |
| Category | 2005-2012 n (%) | 2013-2020 n (%) | 2021-2024 n (%) |
| Cerebrovascular disease | 8,756 (11.4) | 6,910 (9.1) | 2,891 (7.5) |
| Respiratory disease | 11,537 (15.0) | 21,442 (28.3) | — |
| Malignancy | 5,120 (6.7) | 5,053 (6.7) | 2,660 (6.9) |
| Aortic disease | — | — | 4,974 (12.9) |
| Other internal cause | — | — | 9,881 (25.6) |
| External cause (unspecified) | 29,833 (38.9) | 8,776 (11.6) | — |
| Asphyxia | — | — | 9,594 (24.8) |
| Hanging | — | — | 991 (2.6) |
| Traffic injury | — | 5,024 (6.6) | 2,209 (5.7) |
| Other trauma | — | — | 1,531 (4.0) |
| Drowning | — | 1,359 (1.8) | 702 (1.8) |
| Poisoning | — | 219 (0.3) | 114 (0.3) |
| Hypothermia | — | 283 (0.4) | 165 (0.4) |
| Anaphylaxis | — | 99 (0.1) | 43 (0.1) |
| Other external cause | — | — | 2,879 (7.5) |
| Other | 21,327 (27.8) | 26,712 (35.2) | — |
| Not selected | 123 (0.2) | — | — |
| Subtype codes are shown as recorded, without harmonisation across coding eras; denominators are all arrests of non-cardiac origin within each era, and — indicates that the category does not exist in that coding scheme. | | | |
| Asphyxia was recorded under external cause until 2012, under respiratory disease from 2013, and under a dedicated code from 2021. | | | |
