# Supplementary material for: Prehospital predictors of clinical outcomes in out-of-hospital cardiac arrest with pulseless electrical activity: a JCS-ReSS report
Source: Resusc Plus. 2026 Sep 28;32:101508. doi: 10.1016/j.resplu.2026.101508 (PMC13676978; doi:10.1016/j.resplu.2026.101508)
Supplement: Supplementary file 11 [file mmc5.docx]

**Table S1. Prehospital predictors of a favourable neurological outcome, estimated separately among patients with a cardiac and a non-cardiac aetiology**

| Variable | Cardiac RR (95% CI) | Non-cardiac RR (95% CI) | Non-cardiac / cardiac ratio of RR (95% CI) | Non-cardiac minus cardiac difference in RD % points (95% CI) |
| --- | --- | --- | --- | --- |
| **Model A: circumstances at the scene** |  |  |  |  |
| Age, 80 vs 60 years | 0.38 (0.36 to 0.41) | 0.78 (0.72 to 0.85) | 2.04 (1.83 to 2.28) | 2.29 (1.97 to 2.60) |
| Male | 1.20 (1.13 to 1.27) | 0.97 (0.91 to 1.04) | 0.81 (0.75 to 0.89) | -0.44 (-0.61 to -0.26) |
| Witnessed | 1.77 (1.67 to 1.88) | 1.43 (1.34 to 1.54) | 0.81 (0.74 to 0.89) | -0.47 (-0.63 to -0.30) |
| Bystander CPR | 1.02 (0.96 to 1.07) | 0.89 (0.83 to 0.95) | 0.88 (0.81 to 0.95) | -0.27 (-0.44 to -0.10) |
| Response time, 10 vs 5 min | 0.67 (0.63 to 0.71) | 0.65 (0.60 to 0.70) | 0.97 (0.87 to 1.07) | 0.03 (-0.19 to 0.25) |
| **Calendar period (vs 2005-2009)** |  |  |  |  |
| 2010-2014 | 1.48 (1.36 to 1.61) | 1.46 (1.33 to 1.61) | 0.99 (0.87 to 1.13) | -0.05 (-0.26 to 0.17) |
| 2015-2019 | 1.89 (1.74 to 2.05) | 1.83 (1.67 to 2.01) | 0.97 (0.86 to 1.10) | -0.12 (-0.35 to 0.12) |
| 2020-2024 | 1.79 (1.65 to 1.95) | 1.64 (1.48 to 1.81) | 0.91 (0.80 to 1.04) | -0.25 (-0.48 to -0.01) |
| **Model B: adding the intra-arrest course** |  |  |  |  |
| Age, 80 vs 60 years | 0.44 (0.41 to 0.47) | 0.81 (0.75 to 0.89) | 1.86 (1.67 to 2.08) | 1.79 (1.52 to 2.06) |
| Male | 1.12 (1.07 to 1.19) | 1.05 (0.99 to 1.12) | 0.94 (0.86 to 1.02) | -0.15 (-0.32 to 0.02) |
| Witnessed | 1.41 (1.34 to 1.49) | 1.26 (1.18 to 1.35) | 0.89 (0.82 to 0.97) | -0.27 (-0.43 to -0.10) |
| Bystander CPR | 0.88 (0.84 to 0.92) | 0.74 (0.69 to 0.78) | 0.84 (0.78 to 0.91) | -0.32 (-0.49 to -0.16) |
| Response time, 10 vs 5 min | 0.81 (0.76 to 0.86) | 0.76 (0.70 to 0.82) | 0.94 (0.85 to 1.04) | -0.07 (-0.28 to 0.14) |
| Physician-staffed ACLS | 0.86 (0.80 to 0.93) | 0.90 (0.82 to 0.99) | 1.05 (0.93 to 1.18) | 0.11 (-0.11 to 0.34) |
| Conversion to shockable rhythm | 1.37 (1.28 to 1.47) | 0.73 (0.59 to 0.91) | 0.53 (0.43 to 0.67) | -1.32 (-1.70 to -0.94) |
| Prehospital ROSC | 26.54 (24.90 to 28.28) | 20.39 (18.83 to 22.08) | 0.77 (0.69 to 0.85) | -4.58 (-5.11 to -4.04) |
| Post-arrival interval, 30 vs 20 min | 0.66 (0.63 to 0.70) | 0.69 (0.65 to 0.73) | 1.04 (0.96 to 1.12) | 0.15 (-0.02 to 0.32) |
| **Calendar period (vs 2005-2009)** |  |  |  |  |
| 2010-2014 | 1.09 (1.01 to 1.19) | 1.19 (1.09 to 1.31) | 1.09 (0.96 to 1.24) | 0.14 (-0.09 to 0.37) |
| 2015-2019 | 1.25 (1.16 to 1.35) | 1.40 (1.27 to 1.53) | 1.12 (0.99 to 1.26) | 0.18 (-0.06 to 0.41) |
| 2020-2024 | 1.22 (1.12 to 1.32) | 1.32 (1.19 to 1.45) | 1.08 (0.95 to 1.23) | 0.10 (-0.14 to 0.34) |
| Values are risk ratios (95% confidence intervals); the ratio divides the non-cardiac by the cardiac risk ratio, and the difference subtracts the cardiac from the non-cardiac risk difference. | | | | |
| The cohort comprised 251,423 patients with a cardiac and 191,207 with a non-cardiac aetiology. | | | | |
| ACLS, advanced cardiovascular life support; CI, confidence interval; CPR, cardiopulmonary resuscitation; RD, risk difference; RR, risk ratio; ROSC, return of spontaneous circulation. | | | | |
