# Supplementary material for: Prehospital predictors of clinical outcomes in out-of-hospital cardiac arrest with pulseless electrical activity: a JCS-ReSS report
Source: Resusc Plus. 2026 Sep 28;32:101508. doi: 10.1016/j.resplu.2026.101508 (PMC13676978; doi:10.1016/j.resplu.2026.101508)
Supplement: Supplementary file 12 [file mmc6.docx]

**Table S2. Prehospital predictors of 1-month survival, estimated separately among patients with a cardiac and a non-cardiac aetiology**

| Variable | Cardiac RR (95% CI) | Non-cardiac RR (95% CI) | Non-cardiac / cardiac ratio of RR (95% CI) | Non-cardiac minus cardiac difference in RD % points (95% CI) |
| --- | --- | --- | --- | --- |
| **Model A: circumstances at the scene** |  |  |  |  |
| Age, 80 vs 60 years | 0.55 (0.53 to 0.58) | 1.00 (0.96 to 1.04) | 1.81 (1.70 to 1.93) | 4.08 (3.57 to 4.59) |
| Male | 1.11 (1.08 to 1.15) | 0.91 (0.88 to 0.94) | 0.82 (0.78 to 0.86) | -1.30 (-1.61 to -0.99) |
| Witnessed | 1.53 (1.48 to 1.59) | 1.44 (1.39 to 1.49) | 0.94 (0.90 to 0.99) | 0.54 (0.25 to 0.84) |
| Bystander CPR | 1.04 (1.01 to 1.07) | 1.12 (1.09 to 1.16) | 1.08 (1.03 to 1.13) | 0.71 (0.40 to 1.01) |
| Response time, 10 vs 5 min | 0.74 (0.71 to 0.78) | 0.67 (0.64 to 0.69) | 0.90 (0.85 to 0.95) | -1.52 (-1.90 to -1.14) |
| **Calendar period (vs 2005-2009)** |  |  |  |  |
| 2010-2014 | 1.30 (1.24 to 1.37) | 1.18 (1.12 to 1.23) | 0.90 (0.84 to 0.97) | 0.00 (-0.41 to 0.41) |
| 2015-2019 | 1.51 (1.43 to 1.59) | 1.23 (1.17 to 1.28) | 0.81 (0.76 to 0.87) | -0.47 (-0.89 to -0.05) |
| 2020-2024 | 1.44 (1.37 to 1.52) | 1.11 (1.06 to 1.16) | 0.77 (0.72 to 0.83) | -1.00 (-1.42 to -0.58) |
| **Model B: adding the intra-arrest course** |  |  |  |  |
| Age, 80 vs 60 years | 0.61 (0.59 to 0.64) | 1.05 (1.00 to 1.09) | 1.71 (1.61 to 1.82) | 3.52 (3.08 to 3.96) |
| Male | 1.06 (1.02 to 1.09) | 0.97 (0.94 to 1.00) | 0.91 (0.88 to 0.95) | -0.55 (-0.83 to -0.27) |
| Witnessed | 1.30 (1.25 to 1.34) | 1.28 (1.24 to 1.33) | 0.99 (0.94 to 1.04) | 0.53 (0.25 to 0.81) |
| Bystander CPR | 0.94 (0.91 to 0.97) | 0.97 (0.95 to 1.00) | 1.04 (0.99 to 1.08) | 0.13 (-0.14 to 0.41) |
| Response time, 10 vs 5 min | 0.86 (0.83 to 0.90) | 0.77 (0.74 to 0.79) | 0.89 (0.84 to 0.94) | -1.25 (-1.60 to -0.91) |
| Physician-staffed ACLS | 0.92 (0.88 to 0.97) | 0.98 (0.94 to 1.02) | 1.06 (1.00 to 1.13) | 0.27 (-0.13 to 0.67) |
| Conversion to shockable rhythm | 1.33 (1.27 to 1.39) | 0.74 (0.67 to 0.81) | 0.55 (0.50 to 0.61) | -3.76 (-4.39 to -3.13) |
| Prehospital ROSC | 12.49 (12.08 to 12.91) | 9.73 (9.42 to 10.05) | 0.78 (0.74 to 0.82) | 1.39 (0.65 to 2.12) |
| Post-arrival interval, 30 vs 20 min | 0.70 (0.67 to 0.72) | 0.72 (0.70 to 0.74) | 1.03 (0.99 to 1.08) | -0.61 (-0.89 to -0.33) |
| **Calendar period (vs 2005-2009)** |  |  |  |  |
| 2010-2014 | 1.05 (1.00 to 1.10) | 1.01 (0.97 to 1.05) | 0.96 (0.90 to 1.02) | -0.18 (-0.59 to 0.22) |
| 2015-2019 | 1.11 (1.06 to 1.16) | 0.99 (0.95 to 1.04) | 0.90 (0.84 to 0.96) | -0.57 (-0.98 to -0.16) |
| 2020-2024 | 1.08 (1.03 to 1.13) | 0.94 (0.90 to 0.98) | 0.87 (0.82 to 0.93) | -0.87 (-1.29 to -0.46) |
| Values are risk ratios (95% confidence intervals); the ratio divides the non-cardiac by the cardiac risk ratio, and the difference subtracts the cardiac from the non-cardiac risk difference. | | | | |
| The cohort comprised 251,423 patients with a cardiac and 191,207 with a non-cardiac aetiology. | | | | |
| ACLS, advanced cardiovascular life support; CI, confidence interval; CPR, cardiopulmonary resuscitation; RD, risk difference; RR, risk ratio; ROSC, return of spontaneous circulation. | | | | |
