# Supplementary material for: Prehospital predictors of clinical outcomes in out-of-hospital cardiac arrest with pulseless electrical activity: a JCS-ReSS report
Source: Resusc Plus. 2026 Sep 28;32:101508. doi: 10.1016/j.resplu.2026.101508 (PMC13676978; doi:10.1016/j.resplu.2026.101508)
Supplement: Supplementary file 13 [file mmc7.docx]

**Table S3. Standardised risk of a favourable neurological outcome and of 1-month survival, by aetiology**

|  | Standardised risk | |  | |
| --- | --- | --- | --- | --- |
| Outcome | Cardiac % | Non-cardiac % | Non-cardiac / cardiac risk ratio (95% CI) | Non-cardiac minus cardiac difference, % points (95% CI) |
| **Model A: circumstances at the scene** |  |  |  |  |
| Favourable neurological outcome | 2.38 | 1.94 | 0.82 (0.78 to 0.85) | -0.44 (-0.53 to -0.35) |
| 1-month survival | 5.61 | 7.55 | 1.35 (1.32 to 1.38) | 1.94 (1.79 to 2.10) |
| **Model B: adding the intra-arrest course** |  |  |  |  |
| Favourable neurological outcome | 2.67 | 1.66 | 0.62 (0.60 to 0.65) | -1.00 (-1.09 to -0.92) |
| 1-month survival | 6.18 | 6.63 | 1.07 (1.05 to 1.10) | 0.45 (0.31 to 0.59) |
| Values are risks (%) standardised to the covariate distribution of the whole cohort (442,630 patients), from models fitted separately within each aetiology. | | | | |
| CI, confidence interval; RD, risk difference; RR, risk ratio. | | | | |
