# Supplementary material for: Prehospital predictors of clinical outcomes in out-of-hospital cardiac arrest with pulseless electrical activity: a JCS-ReSS report
Source: Resusc Plus. 2026 Sep 28;32:101508. doi: 10.1016/j.resplu.2026.101508 (PMC13676978; doi:10.1016/j.resplu.2026.101508)
Supplement: Supplementary file 14 [file mmc8.docx]

**Table S4. Diagnostics for the time-dependent propensity score matching analyses**

|  |  |  |  |  | Times each control was used | | |  |  |
| --- | --- | --- | --- | --- | --- | --- | --- | --- | --- |
| Analysis | Matched pairs | Match rate | Caliper | Mean absolute difference in logit of the propensity score | Median | 90th percentile | Maximum | Unique controls | Maximum absolute SMD after matching |
| **Epinephrine (time-dependent propensity score matching)** |  |  |  |  |  |  |  |  |  |
| Overall (2011-2024) | 109,172 | 1.000 | 0.445 | 6.3e-06 | 1.0 | 4.0 | 35 | 51,792 | 0.054 |
| Cardiac aetiology | 67,151 | 1.000 | 0.446 | 9.0e-06 | 1.0 | 5.0 | 35 | 29,607 | 0.051 |
| Non-cardiac aetiology | 42,021 | 1.000 | 0.449 | 1.7e-05 | 1.0 | 4.0 | 22 | 22,174 | 0.056 |
| 2010-2014 | 21,358 | 1.000 | 0.386 |  |  |  | 21 | 12,452 | 0.058 |
| 2015-2019 | 37,577 | 1.000 | 0.461 |  |  |  | 29 | 17,816 | 0.060 |
| 2020-2024 | 50,237 | 1.000 | 0.507 |  |  |  | 35 | 21,547 | 0.049 |
| **Advanced airway management (time-dependent propensity score matching)** |  |  |  |  |  |  |  |  |  |
| Overall, any device (2021-2024) | 50,753 | 1.000 | 0.284 | 1.2e-05 | 1.0 | 3.0 | 17 | 29,740 | 0.082 |
| Cardiac aetiology | 32,091 | 1.000 | 0.288 | 1.6e-05 | 1.0 | 3.0 | 17 | 17,896 | 0.082 |
| Non-cardiac aetiology | 18,662 | 1.000 | 0.279 | 3.4e-05 | 1.0 | 3.0 | 13 | 11,824 | 0.075 |
| Endotracheal intubation | 9,672 | 1.000 | 0.314 | 2.8e-05 | 1.0 | 2.0 | 6 | 8,438 | 0.080 |
| Extraglottic airway | 38,699 | 1.000 | 0.288 | 1.1e-05 | 1.0 | 3.0 | 13 | 24,840 | 0.082 |
| Laryngeal mask airway | 2,382 | 1.000 | 0.259 | 1.7e-05 | 1.0 | 1.0 | 4 | 2,300 | 0.100 |
| **Advanced airway management, by aetiology and device** |  |  |  |  |  |  |  |  |  |
| Cardiac, laryngeal mask airway | 1,499 | 1.000 | 0.247 | 2.5e-05 | 1.0 | 1.0 | 4 | 1,437 | 0.114 |
| Cardiac, extraglottic airway | 25,458 | 1.000 | 0.299 | 2.0e-05 | 1.0 | 3.0 | 13 | 15,390 | 0.085 |
| Cardiac, endotracheal intubation | 5,134 | 1.000 | 0.300 | 3.3e-05 | 1.0 | 2.0 | 4 | 4,569 | 0.066 |
| Non-cardiac, laryngeal mask airway | 883 | 1.000 | 0.291 | 8.2e-05 | 1.0 | 1.0 | 2 | 864 | 0.069 |
| Non-cardiac, extraglottic airway | 13,241 | 1.000 | 0.270 | 2.9e-05 | 1.0 | 2.0 | 10 | 9,420 | 0.069 |
| Non-cardiac, endotracheal intubation | 4,538 | 1.000 | 0.233 | 5.9e-05 | 1.0 | 2.0 | 6 | 3,866 | 0.083 |
| Values are medians over 30 imputed datasets; the balance column is the largest absolute standardised mean difference across all columns of the propensity score design matrix after matching. | | | | | | | | | |
| Matching was with replacement, so the number of distinct controls is smaller than the number of matched pairs. | | | | | | | | | |
| Cells are empty for the period-specific epinephrine analyses because those summaries were not produced in that step. | | | | | | | | | |
| EMS, emergency medical services; SMD, standardised mean difference. | | | | | | | | | |
