# Supplementary material for: Prehospital predictors of clinical outcomes in out-of-hospital cardiac arrest with pulseless electrical activity: a JCS-ReSS report
Source: Resusc Plus. 2026 Sep 28;32:101508. doi: 10.1016/j.resplu.2026.101508 (PMC13676978; doi:10.1016/j.resplu.2026.101508)
Supplement: Supplementary file 15 [file mmc9.docx]

**Table S5. Covariate balance for epinephrine before and after time-dependent propensity score matching**

| Covariate | Before matching | After matching |
| --- | --- | --- |
| Age | 0.101 | 0.028 |
| Male | 0.067 | 0.002 |
| Witnessed | 0.047 | 0.010 |
| Bystander CPR | 0.082 | 0.009 |
| Response time | 0.147 | 0.039 |
| Physician-staffed ACLS | 0.022 | 0.054 |
| Conversion to shockable rhythm | 0.219 | 0.032 |
| Non-cardiac aetiology | 0.101 | 0.009 |
| Calendar period | 0.258 | 0.002 |
| Values are absolute standardised mean differences between treated and control person-minutes, pooled over 30 imputed datasets. | | |
| The 2005-2009 period is absent because the time of epinephrine administration was not recorded then; calendar period is contrasted against 2010-2014. | | |
| For covariates represented by several columns (Age, 4 columns; Response time, 4 columns; Calendar period, 2 columns), the largest absolute difference across those columns is shown. | | |
| ACLS, advanced cardiovascular life support; CPR, cardiopulmonary resuscitation; SMD, standardised mean difference. | | |
